# Vitamin D and cause-specific vascular disease and mortality: a Mendelian randomisation study involving 99,012 Chinese and 106,911 European adults

**DOI:** 10.1186/s12916-019-1401-y

**Published:** 2019-08-30

**Authors:** Tao Huang, Shoaib Afzal, Canqing Yu, Yu Guo, Zheng Bian, Ling Yang, Iona Y. Millwood, Robin G. Walters, Yiping Chen, Ningyu Chen, Ruqin Gao, Junshi Chen, Robert Clarke, Zhengming Chen, Christina Ellervik, Børge G. Nordestgaard, Jun Lv, Liming Li, on behalf of the China Kadoorie Biobank Collaborative Group, Junshi Chen, Zhengming Chen, Robert Clarke, Rory Collins, Yu Guo, Liming Li, Jun Lv, Richard Peto, Robin Walters, Daniel Avery, Ruth Boxall, Derrick Bennett, Yumei Chang, Yiping Chen, Zhengming Chen, Robert Clarke, Huaidong Du, Simon Gilbert, Alex Hacker, Mike Hill, Michael Holmes, Andri Iona, Christiana Kartsonaki, Rene Kerosi, Ling Kong, Om Kurmi, Garry Lancaster, Sarah Lewington, Kuang Lin, John McDonnell, Iona Millwood, Qunhua Nie, Jayakrishnan Radhakrishnan, Paul Ryder, Sam Sansome, Dan Schmidt, Paul Sherliker, Rajani Sohoni, Becky Stevens, Iain Turnbull, Robin Walters, Jenny Wang, Lin Wang, Neil Wright, Ling Yang, Xiaoming Yang, Zheng Bian, Yu Guo, Xiao Han, Can Hou, Jun Lv, Pei Pei, Chao Liu, Yunlong Tan, Canqing Yu, Zengchang Pang, Ruqin Gao, Shanpeng Li, Shaojie Wang, Yongmei Liu, Ranran Du, Yajing Zang, Liang Cheng, Xiaocao Tian, Hua Zhang, Yaoming Zhai, Feng Ning, Xiaohui Sun, Feifei Li, Silu Lv, Junzheng Wang, Wei Hou, Mingyuan Zeng, Ge Jiang, Xue Zhou, Liqiu Yang, Hui He, Bo Yu, Yanjie Li, Qinai Xu, Quan Kang, Ziyan Guo, Dan Wang, Ximin Hu, Jinyan Chen, Yan Fu, Zhenwang Fu, Xiaohuan Wang, Min Weng, Zhendong Guo, Shukuan Wu, Yilei Li, Huimei Li, Zhifang Fu, Ming Wu, Yonglin Zhou, Jinyi Zhou, Ran Tao, Jie Yang, Jian Su, Fang Liu, Jun Zhang, Yihe Hu, Yan Lu, Liangcai Ma, Aiyu Tang, Shuo Zhang, Jianrong Jin, Jingchao Liu, Zhenzhu Tang, Naying Chen, Ying Huang, Mingqiang Li, Jinhuai Meng, Rong Pan, Qilian Jiang, Jian Lan, Yun Liu, Liuping Wei, Liyuan Zhou, Ningyu Chen, Ping Wang, Fanwen Meng, Yulu Qin, Sisi Wang, Xianping Wu, Ningmei Zhang, Xiaofang Chen, Weiwei Zhou, Guojin Luo, Jianguo Li, Xiaofang Chen, Xunfu Zhong, Jiaqiu Liu, Qiang Sun, Pengfei Ge, Xiaolan Ren, Caixia Dong, Hui Zhang, Enke Mao, Xiaoping Wang, Tao Wang, Xi Zhang, Ding Zhang, Gang Zhou, Shixian Feng, Liang Chang, Lei Fan, Yulian Gao, Tianyou He, Huarong Sun, Pan He, Chen Hu, Xukui Zhang, Huifang Wu, Pan He, Min Yu, Ruying Hu, Hao Wang, Yijian Qian, Chunmei Wang, Kaixu Xie, Lingli Chen, Yidan Zhang, Dongxia Pan, Qijun Gu, Yuelong Huang, Biyun Chen, Li Yin, Huilin Liu, Zhongxi Fu, Qiaohua Xu, Xin Xu, Hao Zhang, Huajun Long, Xianzhi Li, Libo Zhang, Zhe Qiu

**Affiliations:** 1 Department of Epidemiology and Biostatistics, School of Public Health, Peking University Health Science Center, 38 Xueyuan Road, Beijing, 100191 China; 2 Key Laboratory of Molecular Cardiovascular Sciences (Peking University), Ministry of Education, Beijing, China; 3 Department of Clinical Biochemistry and Copenhagen General Population Study, Copenhagen University Hospital, Herlev and Gentofte Hospital Copenhagen, Copenhagen, Denmark; 4 Chinese Academy of Medical Sciences, Beijing, China; 5 Clinical Trial Service Unit & Epidemiological Studies Unit (CTSU), Nuffield Department of Population Health, University of Oxford, Oxford, UK; 6 NCDs Prevention and Control Department, Liuzhou Center for Disease Control and Prevention, Liuzhou, China; 7 Qingdao Center for Disease Control and Prevention, Qingdao, China; 8 China National Center for Food Safety Risk Assessment, Beijing, China; 9 The Copenhagen City Heart Study, Frederiksberg Hospital, Copenhagen University Hospital, Copenhagen, Denmark; 10 Faculty of Health and Medical Sciences, University of Copenhagen, Copenhagen, Denmark; 11 Peking University Institute of Environmental Medicine, Beijing, China

**Keywords:** Mendelian randomisation, Vitamin D, Cardiovascular diseases, Lipids, Causal effect

## Abstract

**Background:**

Randomised control trials and genetic analyses have demonstrated that vitamin D or 25-hydroxyvitamin D (25[OH]D) levels may not play a causal role in the development of cardiovascular disease. However, it is unclear if 25(OH)D is causally associated with cause-specific vascular disease and lipids. Therefore, we examined the causal association of 25(OH)D with myocardial infarction, stroke, ischaemic heart disease, ischaemic stroke, subarachnoid haemorrhage, intracerebral haemorrhage, and lipid levels among both Chinese and Europeans.

**Methods:**

We used a Mendelian randomisation (MR) design in the China Kadoorie Biobank, the Copenhagen City Heart Study, and the Copenhagen General Population Study. The 25(OH)D-related genetic variants in the *CYP2R1* and *DCHR7* genes were genotyped in 99,012 Chinese adults and 106,911 Danish adults.

**Results:**

In Chinese adults, plasma 25(OH)D levels were not significantly associated with cause-specific vascular disease or mortality, with the exception of intracerebral haemorrhage (HR, 1.09 [95% CI, 1.01,1.18] per 25 nmol/L higher plasma 25(OH)D). In Europeans, plasma 25(OH)D levels were inversely associated with all types of vascular diseases and mortality. However, MR analysis did not demonstrate causal associations of genetically increased 25(OH)D levels with cause-specific vascular diseases, or mortality in both Chinese and European adults. In addition, each 25 nmol/L higher 25(OH)D was observationally associated with lower total cholesterol and low-density lipoprotein cholesterol levels, but higher high-density lipoprotein cholesterol levels. Likewise, MR analysis showed that 25(OH)D levels were not causally associated with lipids in both Chinese and European adults after Bonferroni correction.

**Conclusions:**

We found no evidence to support that genetically increased 25(OH)D was associated with a lower risk of ischaemic stroke, intracerebral haemorrhage, subarachnoid haemorrhage, and lipid levels in both Chinese and European adults. These results suggest that the inverse associations of vitamin D with vascular disease could be the result of confounding.

**Electronic supplementary material:**

The online version of this article (10.1186/s12916-019-1401-y) contains supplementary material, which is available to authorized users.

## Background

Observational studies have shown that low plasma levels of vitamin D are associated with higher risks of cardiovascular disease (CVD) [1–3] and mortality from CVD, cancer, and all causes [2, 4, 5] in European populations. However, randomised intervention trials (RCTs) of vitamin D3 (cholecalciferol) supplementation have not clearly demonstrated any protective effect on CVD [6–9] or all-cause mortality [10, 11]. Findings from Mendelian randomisation (MR) analysis also did not indicate causal association of 25-hydroxyvitamin D (25[OH]D), usually used to assess vitamin D status, with increased risk of ischaemic heart disease, myocardial infarction [12], and cardiovascular mortality [13], but showed a possible effect on all-cause and cancer mortality in Europeans [13].

No study has evaluated these associations in the Chinese population, while two studies examined risk of diabetes [14, 15]. Importantly, the causal associations between 25(OH)D and cause-specific vascular disease such as stroke, ischaemic stroke, intracerebral haemorrhage, subarachnoid haemorrhage, and lipid levels have not been examined among European and Chinese adults. Whether a high 25(OH)D level is a cause of decreased vascular disease and mortality or simply a consequence of poor health is thus unclear. This question has important public health relevance, as about half of the population have vitamin D deficiency and regularly take vitamin D supplements [16], but without proper scientific evidence for any beneficial effect on many disease outcomes.

With the lack of evidence from intervention trials, a MR approach, which is regarded as a natural RCT, can potentially be used to make causal inference [17–22]. MR uses the random assortment of genes during gamete formation as the natural randomisation process. Therefore, genetic variants known to influence 25(OH)D concentrations can be used to examine causality as they partly determine lifetime exposure to 25(OH)D. Genome-wide association studies (GWAS) have identified several single nucleotide polymorphisms (SNPs) that are associated with plasma 25(OH)D concentration in relation to genes involved in the synthesis of 25(OH)D (*DHCR7* and *CYP2R1*), transport of 25(OH)D via vitamin D-binding protein (*GC/DBP*), and metabolism of 25(OH)D (*CYP24A1*) [23, 24].

The aims of the present study were to test whether higher 25(OH)D concentrations are observationally associated with cause-specific vascular diseases, or mortality and lipids, and whether such associations are causal among European and Chinese adults.

## Methods

### Study populations

The China Kadoorie Biobank (CKB) is a prospective study of 512,891 individuals, aged 30–79 years, who were enrolled from 5 urban and 5 rural regions in China during 2004–2008. Previous studies have reported the details of the study design, participant characteristics, and survey methods [21]. Information on demographic data, lifestyle, use of medications, and medical history were collected by trained health workers by using laptop-based questionnaires. Blood pressure, weight, and height were measured using standard protocols. Body mass index (BMI) was calculated as body weight in kilogrammes divided by height in metres squared. A 10-mL nonfasting venous blood sample was collected. All participants from CKB provided written informed consent. Ethics approval was obtained from the Peking University and University of Oxford ethics committees.

The Copenhagen General Population Study (CGPS) is a prospective cohort study among Danish population initiated in 2003 [25]. The Copenhagen City Heart Study (CCHS) was initiated in 1976–1978, and followed up in 1981–1983, 1991–1994, and 2001–2003 [13]. Body weight and height was measured by standard methods. Blood pressure was measured in millimetres of mercury with a sphygmomanometer or an automated digital blood pressure monitor (Kivex). Leisure time physical activity was considered in four categories. Smoking information was classified as pack-years smoked. Alcohol consumption was based on average weekly consumption of beer, wine, and liquor. Written informed consent was obtained from participants. Ethics approval was obtained from Danish ethical committees.

### Biochemical analysis

In CKB, plasma 25(OH)D concentrations were measured using a Beckman Access-2 immunoassay assay on stored EDTA plasma samples in 13,565 individuals. The laboratory participated in the international Vitamin D Quality Assessment Scheme (DEQAS) for 25(OH)D and results had a mean (SD) bias of − 11.8% (7.5) from the target mean value for the present study. Samples of the CCHS were stored at − 20 °C (1981–1983), and samples from the CGPS were stored at − 80 °C (2004–2005). Colorimetric assays (Mannheim, Boehringer Mannheim, Germany or Konelab, Finland, Espoo) were used to measure plasma high-density lipoprotein (HDL) cholesterol and total cholesterol concentrations. 25(OH)D concentrations were measured in both serum and plasma using a chemiluminescent immunoassay, CLIA (DiaSorin, Stillwater, MN, USA). The inter- and intra-assay coefficients of variation were 11% and 10%, respectively.

### SNP selection and genotyping

A total of 95,680 individuals from the CKB were genotyped using a 384-SNP panel (Illumina Golden Gate). Two synthesis SNPs (*CYP2R1*-rs10741657 and *DHCR7*-rs12785878), one transport SNP (*GC/DBP-*rs2282679), and one metabolism SNP (*CYP24A1*-rs6013897) identified for plasma 25(OH)D concentrations in previously GWAS were included in the SNP panel [20, 21]. The genotyping concordance was > 99.9% for 2063 pairs of sample replicates and genotype success rates were 99.9% for each SNP. In the CGPS, genetic variants for 25(OH)D were selected based on a reported GWAS [23, 24]. The selected SNPs either influence synthesis of pre-vitamin D from 7-dehydrocholesterol in the skin or activate transformation from vitamin D to 25(OH)D in the liver. TaqMan assays were used for genotyping for *DHCR7* (rs7944926 and rs11234027) and *CYP2R1* (rs10741657 and rs12794714). Sequencing of randomly selected samples verified the genotypes. All genotype call rates were higher than 99% complete.

### Outcomes

The present study examined major coronary events (fatal ischaemic heart disease [ICD-10: I20-I25] or nonfatal myocardial infarction [I21-I23]), major vascular events [I60 and I64], intracerebral haemorrhage (I61), ischaemic stroke (I63), and cardiovascular death (I00-I99) as the primary outcomes in the CKB. Data on disease outcomes were identified from claims to the national health insurance system and from local chronic disease registries and disease surveillance point system death registries. Trained staff members, blinded to the baseline information, used International Statistical Classification of Diseases and Related Health Problems (ICD-10) to identify the underlying causes of death. The deaths were grouped into several categories: major coronary events [ICD-10: I20-I25], major vascular events [I60 and I64], ischaemic heart disease (I20-I25), myocardial infarction [I21-I23], ischaemic stroke (I63), intracerebral haemorrhage (I61), cancer (C00-C97), diseases of the respiratory system (J00-J99), infections (A00-B99), and all other causes.

In the CGPS and CCHS studies, information on diagnoses of vascular diseases such as myocardial infarction (ICD-8: 410; ICD-10: I21-I22) and ischaemic heart disease (ICD-8: 410 to 414, and ICD-10: I20 to I25) were verified based on information on diagnoses entered in the national Danish Patient Registry and hospital admissions. The national Danish Causes of Death Registry records were used to identify the causes of death as well as contributing causes of death [12]. We used underlying causes of death to classify deaths as due to cardiovascular (ICD-8 390-458, ICD-10 I00-I99) and cancer (ICD-8 140-209, ICD-10 C00-C97) [13].

### Statistical methods

For observational analyses, data were available on 13,565 participants with plasma 25(OH)D levels for observational analyses, and data were available on 82,464 participants with all genotypes. Of these, 3397 had data on both 25(OH)D plasma levels and genotypes in CKB. We included 25,621 participants and 10,271 participants with plasma 25(OH)D levels in CGPS and CCHS, respectively; in genetic analyses, we included 106,911 participants with all genotypes. Of these, 25,332 had both 25(OH)D plasma levels and genotypes in CGPS. Cox proportional hazard regression models were used to analysing the relationship between quartile groups of 25(OH)D levels and risk of disease. We used Schoenfeld residual test to examine the proportional hazard assumption. All results (*P* > 0.05) showed that the Cox models were acceptable. Multivariable Cox regression analyses included age (years), sex (male or female), smoking status (current smoker), alcohol intake (current drinker), season, region, systolic blood pressure (SBP), physical activity (low activity) in the CKB, and age (years), sex (male or female), season, SBP, physical activity (low activity), smoking status (current smoker), and alcohol intake (current drinker) in the CGPS and CCHS.

Genotype distributions in each study did not differ from the Hardy-Weinberg equilibrium. Linear regression was used to examine the per allele effects of each SNP on plasma 25(OH)D concentrations. Genetic scores were estimated for the two synthesis SNPs and all four SNPs. The selected SNPs are not in linkage disequilibrium (LD). F-statistics were used to estimate the strength of associations of each SNP with 25(OH)D concentrations, and F-statistics > 10 were considered strong. We also used linear regression to assess the relationship of each SNP with plasma lipids and lipoproteins. All genetic analyses were adjusted for age at baseline, sex, season, and region. Analyses of individual SNPs and genetic scores with outcomes were estimated using Cox proportional hazard regression models. The per allele effects of each SNP on plasma 25(OH)D concentrations were expressed as the differences in 25(OH)D concentrations per copy of the 25(OH)D raising allele.

A two-stage least squared regression model using genotype individually and combined allele scores as instruments was used to examine the influence of a 25 nmol/L increase in 25(OH)D concentrations on risk of outcomes. We used the *DHCR7*/*CYP2R1* genetic score as the main instrumental variable. We calculated causal estimates of genetically determined hazard ratios (HRs) by using the Wald-type estimator, which calculates the ratio of the outcome allele score log hazard ratios to the exposure allele score coefficient and then exponentiates the ratio to a hazard ratio. For combination of CVD and lipid data from Asians and Europeans, meta-analyses were conducted. We assessed between-study heterogeneity via Cochrane’s *Q*- and *I*^2^-statistics. A random-effect meta-analysis was used if *I*^2^ > 0.25, otherwise, a fixed-effect model was used [26].

Sensitivity analyses were performed for three-SNP and four-SNP genetic scores to examine the potential effects of pleiotropy using the MR-Egger regression method [27]. We used SAS version 9.2 and R version 3.01. We included 20 diseases or cause-specific mortality as primary outcomes. The threshold of significance was *P* < 0.0025 (0.05/20) after Bonferroni correction. *P* ≤ 0.05 but above the threshold of Bonferroni corrected significance was considered as a suggestive causal association.

## Results

### Characteristics of the CKB, CGPS, and CCHS populations

Among the 82,464 CKB individuals and 106,911 CGPS individuals in the genetic study, the mean (SD) of age was 51.4 (10.6) and 58.0 (13.1) years, 61% and 55% were women, and the mean (SD) of BMI was 23.7 (3.4) and 26.1 (4.3) kg/m^2^, respectively. The overall mean (SD) plasma 25(OH)D concentrations were 63.7 (26.6) nmol/L, 55.3 (26.0) nmol/L, 44.3 (24.1) nmol/L in CKB, CGPS, and CCHS, respectively (Table 1).

**Table 1:** Characteristics for all individuals with 25(OH)D measured or with genetic data

Baseline	All individuals with 25(OH)D measured			Individuals with genetic data	
Characteristic	CKB	CGPS	CCHS	CKB	CGPS
Demographic	(*n* = 13,565)	(*n* = 25,621)	(*n* = 10,271)	(*n* = 82,464)	(*n* = 106,911)
Age (SD), years	53.2 (11.2)	58.5 (13.0)	56.7 (11.9)	51.4 (10.6)	58.0 (13.1)
Women, %	49.2	54.9	55.7	60.5	55
Current smoker, %	47.2	20.5	58.4	36.9	17.1
Current drinker, %	56.8	88.3	69	53.7	89.1
Low physical activity^*^	18.15	6.8	16.8	18.15	6.2
BMI (SD), kg/m^2^	23.6 (3.5)	26.1 (4.3)	25.3 (4.2)	23.7 (3.4)	26.1 (4.3)
SBP (SD), mmHg	140.2 (25.9)	141.1 (21.1)	140.5 (21.6)	131.2 (21.3)	141.6 (21.4)
DBP (SD), mmHg	82.2 (14.5)	83.9 (11.4)	85.1 (12.1)	77.8 (11.2)	84.3 (11.5)
Doctor diagnosed prior disease, %					
Heart disease	0	6.1	3.5	3	5.8
Stroke	0	1.4	1	1.8	1.3
Hypertension	15.9	60.9	56.1	11.5	60.3
Diabetes	3.7	4.5	3.3	3.2	4.2
Cancer	0	6.5	4	0.5	6.9
Current medication, %					
Statin use	0	11.6	NA	0.2	12.1
Aspirin use	1.3	12.7	NA	1.1	11.7
Blood pressure lowering	6.1	20.3	10.8	4.8	19.8
Plasma 25(OH)D (SD), nmol/L	83.7 (26.6)	55.3 (26.0)	44.3 (24.1)	83.4 (26.2)	55.3 (26.1)

*CKB* the China Kadoorie Biobank, *CGPS* the Copenhagen General Population Study, *CCHS* the Copenhagen City Heart Study, *SD* standard deviation, *BMI* body mass index, *SBP* systolic blood pressure, *DBP* diastolic blood pressure

^*^Self-reported passivity or less than 2 h of light physical activity a week in CGPS and CCHS; MET-h/day < 8.4 (the bottom quintile) in CKB

A total of 3397 individuals in the genetic study in CKB had plasma 25(OH)D concentrations measured

25(OH)D: 1 ng/ml = 2.496 nmol/L

### Observational associations

In CKB, plasma 25(OH)D levels were not statistically associated with major vascular events (multivariable adjusted HR per 25 nmol/L higher plasma 25(OH)D, 1.02 [95% CI, 0.98,1.07]), major coronary events (HR, 0.98 [95% CI, 0.89,1.07]), myocardial infarction (HR, 0.99 [95% CI, 0.89,1.12]), stroke (HR, 1.04 [95% CI, 0.99,1.10]), ischaemic stroke (HR, 0.99[95% CI, 0.93,1.07]), subarachnoid haemorrhage (HR, 1.24 [95% CI, 0.83,1.85]), and ischaemic heart disease (HR, 0.98 [95% CI, 0.89,1.07]) after adjustment for age, sex, smoking status, alcohol intake, season, region, SBP, and physical activity. However, each 25 nmol/L higher plasma 25(OH)D concentration was associated with about a 9% higher risk of intracerebral haemorrhage (HR, 1.09 [95% CI, 1.01,1.18]) (Table 2). Likewise, plasma 25(OH)D levels were not significantly associated with cancer, respiratory diseases, infection-cause mortality, or all-cause mortality after adjustment for confounding factors (Table 3). In addition, each 25 nmol/L higher plasma 25(OH)D concentration was associated with a 1.52 mg/dL higher apoA, 0.04 mg/dL higher apoB, and 0.03 mmol/L (1 mg/dL) higher HDL cholesterol, but a 0.05 mmol/L (2 mg/dL) lower total cholesterol, 0.03 mmol/L (1 mg/dL) lower LDL cholesterol, and a 0.16 mmol/L (14 mg/dL) lower triglycerides (Table 4).

**Table 2:** Observational association of 25(OH)D with risk of vascular diseases in CKB, CGPS, and CCHS

Diseases	Continuous 25(OH)D per 25 nmol/L		25(OH)D quartiles (nmol/L)				*P* for trend
	No. of all events	HR (95% CI)	Quartile 1	Quartile 2	Quartile 3	Quartile 4	
			HR (95% CI)	HR (95% CI)	HR (95% CI)	HR (95% CI)	
CKB							
Major vascular event	3868	1.02(0.98,1.07)	1.00	1.00(0.89,1.12)	1.08(0.95,1.22)	1.03(0.90,1.18)	0.344
Major coronary event	1024	0.98(0.89,1.07)	1.00	0.89(0.71,1.13)	0.99(0.78,1.27)	0.85(0.66,1.12)	0.614
Myocardial infarction	662	0.99(0.89,1.12)	1.00	0.94(0.69,1.27)	1.05(0.77,1.43)	0.99(0.71,1.38)	0.925
Stroke	2982	1.04(0.99,1.10)	1.00	1.01(0.89,1.15)	1.08(0.94,1.24)	1.08(0.93,1.26)	0.154
Ischaemic stroke	1776	0.99(0.93,1.07)	1.00	0.95(0.81,1.11)	1.04(0.88,1.23)	0.96(0.79,1.16)	0.882
Intracerebral haemorrhage	1308	1.09(1.01,1.18)	1.00	1.04(0.84,1.29)	1.08(0.86,1.35)	1.19(0.93,1.51)	0.029
Subarachnoid haemorrhage	56	1.24(0.83,1.85)	1.00	1.05(0.37,2.97)	0.42(0.10,1.72)	1.38(0.42,4.54)	0.291
Ischaemic heart disease	1024	0.98(0.89,1.07)	1.00	0.90(0.71,1.15)	1.04(0.81,1.32)	0.84(0.64,1.10)	0.596
CGPS and CCHS							
Cardiovascular disease	12,110	0.90(0.88,0.93)	1.00	0.92(0.86,0.98)	0.85(0.79,0.91)	0.79(0.73,0.86)	1.3 × 10^−9^
Myocardial infarction	4316	0.88(0.84,0.93)	1.00	0.86(0.77,0.96)	0.81(0.72,0.91)	0.74(0.65,0.85)	5.8 × 10^−10^
Stroke	4490	0.93(0.90,0.98)	1.00	0.97(0.87,1.08)	0.87(0.77,0.98)	0.89(0.78,1.02)	0.022
Ischaemic stroke	3766	0.93(0.88,0.98)	1.00	0.98(0.87,1.11)	0.84(0.73,0.96)	0.90(0.78,1.04)	0.028
Intracerebral haemorrhage	542	1.05(0.90,1.19)	1.00	0.96(0.69,1.33)	1.14(0.82,1.61)	0.96(0.65,1.41)	0.87
Ischaemic heart disease	9362	0.90(0.86,0.93)	1.00	0.91(0.84,0.98)	0.86(0.79,0.93)	0.77(0.70,0.84)	1.4 × 10^−11^

Multivariable cox proportional hazard regression models were used to examine the association between quartile groups of 25(OH)D concentrations and risk of vascular diseases

All values are adjusted for age (years), sex (male or female), smoking status (current smoker), alcohol intake (current drinker), season, region, SBP, and physical activity (low activity) in CKB

All values are adjusted for age (years), sex (male or female), latitude, season, SBP, physical activity (METs, h/day), smoking status (never smoker, occasional smoker, former smoker, or regular smoker), and alcohol intake (non-drinker, occasional drinker, former drinker, or regular drinker) in CGPS and CCHS

**Table 3:** Observational association of 25(OH)D with risk of mortality in the CKB, CGPS, and CCHS

Mortality	Continuous 25(OH)D per 25 nmol/L		25(OH)D quartiles (nmol/L)				*P* for trend
	No. of all events	HR (95% CI)	Quartile 1	Quartile 2	Quartile 3	Quartile 4	
			HR (95% CI)	HR (95% CI)	HR (95% CI)	HR (95% CI)	
CKB							
All-cause mortality	3868	1.02(0.97,1.06)	1.00	0.95(0.84,1.07)	0.97(0.85,1.10)	0.95(0.83,1.09)	0.458
Vascular death outcomes							
Major vascular event	3048	1.04(0.99,1.09)	1.00	0.93(0.81,1.07)	0.97(0.84,1.12)	1.00(0.86,1.17)	0.158
Major coronary event	1262	1.05(0.97,1.14)	1.00	0.95(0.77,1.18)	0.98(0.79,1.23)	1.13(0.89,1.42)	0.186
Myocardial infarction	898	1.04(0.94,1.14)	1.00	0.90(0.70,1.16)	1.02(0.79,1.33)	1.02(0.77,1.36)	0.427
Stroke	1590	1.06(0.98,1.13)	1.00	0.95(0.78,1.14)	1.01(0.82,1.23)	1.01(0.82,1.26)	0.136
Ischaemic stroke	118	1.06(0.84,1.33)	1.00	0.58(0.30,1.12)	0.61(0.30,1.21)	0.84(0.42,1.67)	0.622
Intracerebral haemorrhage	1416	1.05(0.98,1.14)	1.00	0.97(0.80,1.19)	1.06(0.85,1.32)	1.04(0.82,1.31)	0.166
Subarachnoid haemorrhage	68	1.01(0.98,1.03)	1.00	0.95(0.18,4.93)	1.52(0.32,7.33)	1.10(0.18,6.83)	0.685
Ischaemic heart disease	1262	1.05(0.97,1.14)	1.00	0.95(0.77,1.18)	0.98(0.79,1.23)	1.13(0.89,1.42)	0.186
Non-vascular death outcomes							
Cancer	370	1.00(0.99,1.01)	1.00	1.22(0.81,1.83)	1.17(0.76,1.79)	1.00(0.63,1.58)	0.818
Respiratory diseases	46	1.00(0.99,1.01)	1.00	0.91(0.47,1.73)	1.28(0.68,2.42)	0.75(0.35,1.60)	0.798
Infections	64	1.00(0.98,1.02)	1.00	0.51(0.08,3.17)	0.38(0.06,2.53)	1.00(0.21,4.81)	0.946
CGPS and CCHS							
All-cause mortality	10,845	0.90(0.88,0.93)	1.00	0.81(0.77,0.85)	0.79(0.75,0.83)	0.77(0.73,0.82)	2 × 10^−20^
Vascular death outcomes							
Cardiovascular disease	3303	0.90(0.86,0.93)	1.00	0.85(0.78,0.93)	0.78(0.71,0.87)	0.80(0.72,0.90)	1.9 × 10^−8^
Myocardial infarction	676	0.86(0.78,0.98)	1.00	0.68(0.55,0.83)	0.78(0.63,0.97)	0.70(0.54,0.90)	0.0037
Stroke	743	0.93(0.86,1.03)	1.00	0.97(0.81,1.17)	0.74(0.60,0.92)	1.00(0.79,1.26)	0.28
Ischaemic stroke	385	0.88(0.78,1.00)	1.00	1.06(0.82,1.36)	0.65(0.48,0.89)	0.96(0.69,1.34)	0.18
Intracerebral haemorrhage	178	1.05(0.88,1.25)	1.00	1.06(0.71,1.57)	0.96(0.62,1.49)	1.16(0.72,1.86)	0.71
Ischaemic heart disease	1395	0.88(0.82,0.95)	1.00	0.80(0.70,0.92)	0.80(0.69,0.93)	0.76(0.63,0.90)	6 × 10^−4^
Non-vascular death outcomes							
Cancer	3127	0.93(0.88,0.98)	1.00	0.86(0.78,0.94)	0.87(0.78,0.96)	0.79(0.71,0.89)	9.7 × 10^−5^
Respiratory diseases	1065	0.86(0.80,0.93)	1.00	0.69(0.59,0.82)	0.67(0.56,0.80)	0.71(0.59,0.87)	6.6 × 10^−4^
Infections	430	0.84(0.76,0.95)	1.00	0.76(0.59,0.98)	0.85(0.66,1.11)	0.73(0.53,1.00)	0.082
All other causes	2463	0.86(0.82,0.90)	1.00	0.73(0.66,0.81)	0.71(0.63,0.79)	0.68(0.59,0.78)	4.6 × 10^−10^

Multivariable cox proportional hazard regression models were used to examine the association between quartile groups of 25(OH)D concentrations and risk of mortality

All values are adjusted for age (years), sex (male or female), smoking status (current smoker), alcohol intake (current drinker), season, region, SBP, and physical activity (low activity) in CKB

All values are adjusted for age (years), sex (male or female), latitude, season, SBP, physical activity (METs, h/day), smoking status (never smoker, occasional smoker, former smoker, or regular smoker), and alcohol intake (non-drinker, occasional drinker, former drinker, or regular drinker) in CGPS and CCHS

**Table 4:** Observational association of 25(OH)D with lipids in CKB, CGPS, and CCHS

Lipids	Continuous 25(OH)D per 25 nmol/L	25(OH)D quartiles (nmol/L)				*P* for trend
	Beta ± SE	Quartile 1	Quartile 2	Quartile 3	Quartile 4	
		Mean ± SD	Mean ± SD	Mean ± SD	Mean ± SD	
CKB (*n* = 17,755)						
Apoa, mg/dL	1.52 ± 0.21	127.90 ± 0.43	129.48 ± 0.42	129.79 ± 0.41	130.73 ± 0.45	< 0.0001
Apob, mg/dL	0.04 ± 0.00	84.52 ± 0.46	85.95 ± 0.45	84.14 ± 0.44	81.42 ± 0.44	< 0.0001
Lpa, mmol/L	0.01 ± 0.49	37.94 ± 1.00	36.40 ± 0.99	35.17 ± 0.96	37.15 ± 1.02	0.984
TC, mmol/L	−0.05 ± 0.01	4.65 ± 0.02	4.74 ± 0.02	4.66 ± 0.02	4.53 ± 0.02	< 0.0001
HDL, mmol/L	0.03 ± 0.01	1.20 ± 0.01	1.22 ± 0.01	1.23 ± 0.01	1.26 ± 0.01	< 0.0001
LDL, mmol/L	−0.03 ± 0.01	2.32 ± 0.01	2.38 ± 0.01	2.34 ± 0.01	2.28 ± 0.02	< 0.0001
TG, mmol/L	−0.16 ± 0.02	2.18 ± 0.04	2.09 ± 0.03	2.03 ± 0.03	1.75 ± 0.03	< 0.0001
CGPS and CCHS (*n* = 106,911)						
Apoa, mg/dL*	0.01 ± 0.002	160.63 ± 31.08	161.78 ± 29.96	163.35 ± 30.09	164.39 ± 32.33	< 0.0001
Apob, mg/dL*	−0.08 ± 0.002	125.27 ± 40.06	118.58 ± 35.59	112.93 ± 33.17	104.34 ± 29.31	< 0.0001
Lpa, mg/dL*	0.32 ± 0.231	22.99 ± 31.53	22.80 ± 30.25	22.83 ± 32.66	23.90 ± 31.30	0.15
TC, mmol/L	−0.14 ± 0.006	5.85 ± 1.16	5.80 ± 1.12	5.71 ± 1.10	5.53 ± 1.09	< 0.0001
HDL, mmol/L	0.06 ± 0.003	1.38 ± 0.50	1.46 ± 0.51	1.51 ± 0.51	1.56 ± 0.52	< 0.0001
LDL, mmol/L*	−0.15 ± 0.006	3.44 ± 1.00	3.36 ± 0.96	3.27 ± 0.95	3.09 ± 0.91	< 0.0001
TG, mmol/L*	−0.26 ± 0.007	2.08 ± 1.49	1.78 ± 1.10	1.60 ± 0.95	1.39 ± 0.77	< 0.0001

Linear regression was also used to assess the associations of 25(OH)D with lipids

All values are adjusted for age (years), sex (male or female), smoking status (current smoker), alcohol intake (current drinker), season, SBP, and physical activity (low activity) in CKB

All values are adjusted for age (years), sex (male or female), latitude, season, region, SBP, physical activity (METs, h/day), smoking status (never smoker, occasional smoker, former smoker, or regular smoker), and alcohol intake (non-drinker, occasional drinker, former drinker, or regular drinker) in CGPS and CCHS

*Data only available in CGPS

In CGPS and CCHS, each 25 nmol/L higher plasma 25(OH)D concentration was associated with about a 10% lower risk of CVD (multivariable adjusted HR, 0.90 [95% CI, 0.88,0.93]), 12% lower risk of myocardial infarction (HR, 0.88 [95% CI, 0.84,0.93]), 7% lower risk of stroke (HR, 0.93 [95% CI, 0.90,0.98]), 7% lower risk of ischaemic stroke (HR, 0.93 [95% CI, 0.88,0.98]), and 10% lower risk of ischaemic heart disease (HR, 0.90 [95% CI, 0.86,0.93]), but not with intracerebral haemorrhage (HR, 1.05 [95% CI, 0.90,1.19]) (Table 2). Individuals in the top quintile of plasma 25(OH)D concentrations had a 21% lower risk of incident CVD (HR, 0.79 [95% CI, 0.73, 0.86) compared with those in the bottom quintile. For specific types of CVD, the adjusted HR for the top vs bottom quintile of plasma 25(OH)D concentrations were HR 0.74 [95% CI, 0.65,0.85] for myocardial infarction and HR 0.77 [95% CI, 0.70,0.84] for ischaemic heart disease (Table 2). In addition, a 25 nmol/L higher plasma 25(OH)D concentration was associated with all-cause and cause-specific mortality. The multivariable adjusted HR was 0.90 (0.88,0.93) for all-cause mortality, 0.90 (0.86,0.93) for cardiovascular mortality, 0.86 (0.78,0.98) for mortality from myocardial infarction, 0.88 (0.82,0.95) for mortality from ischaemic heart disease, 0.93 (0.88,0.98 for cancer mortality, 0.86 (0.80,0.93) for respiratory disease mortality, and 0.86 (0.82,0.90) for other mortality (Table 3).

### Genetic associations with plasma 25(OH)D and risk factors

**Fig. 1:**
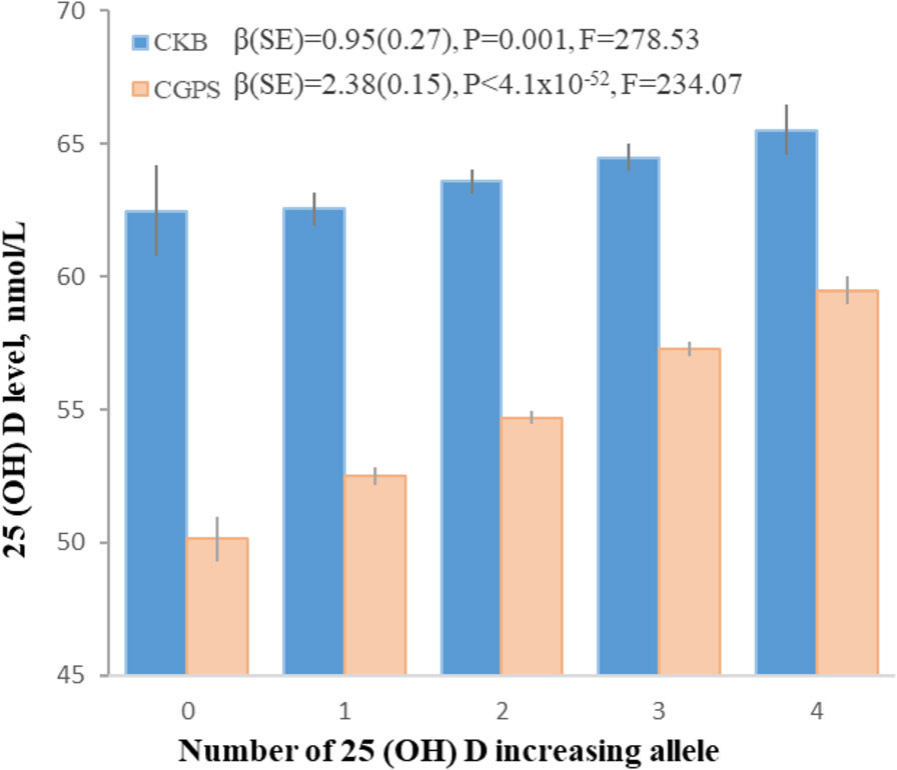
Genetic association with 25(OH)D (nmol/L) in CKB and CGPS. Data are presented as beta ± SE. Linear regression was used to assess the per allele effect of each SNP or genetic score on plasma 25(OH)D concentrations. All values are adjusted for age, sex, and season and stratified by region. CKB, the China Kadoorie Biobank; CGPS, the Copenhagen General Population Study

In CKB, plasma 25(OH)D concentrations per each increasing allele were 1.49 nmol/L (SE 0.38, F-statistic = 89) higher for rs12785878 (*DHCR7*), 0.34 nmol/L (SE 0.15, F-statistic = 97) higher for rs10741657 (*CYP2R1*), 5.10 nmol/L (SE 0.41, F-statistic = 89) higher for rs2282679 (GC/DBP), 0.95 nmol/L (SE 0.27, F-statistic = 278.53) higher for two-SNP score (rs12785878 + rs10741657), and 1.84 nmol/L (SE 0.21, F-statistic = 69) higher for four-SNP score (Fig. 1 and Additional file 1: Table S1). In CGPS, genetic variants and genetic score were also significantly associated with plasma 25(OH)D concentrations (Fig. 1 and Additional file 1: Table S1). None of the SNPs was associated with differences in confounders such as age, gender, season, region, smoking, drinking, physical activity, blood pressure, and BMI in CKB or in CGPS (Additional file 1: Tables S2 & S3).

### Causal associations with cause-specific vascular disease or mortality

**Fig. 2:**
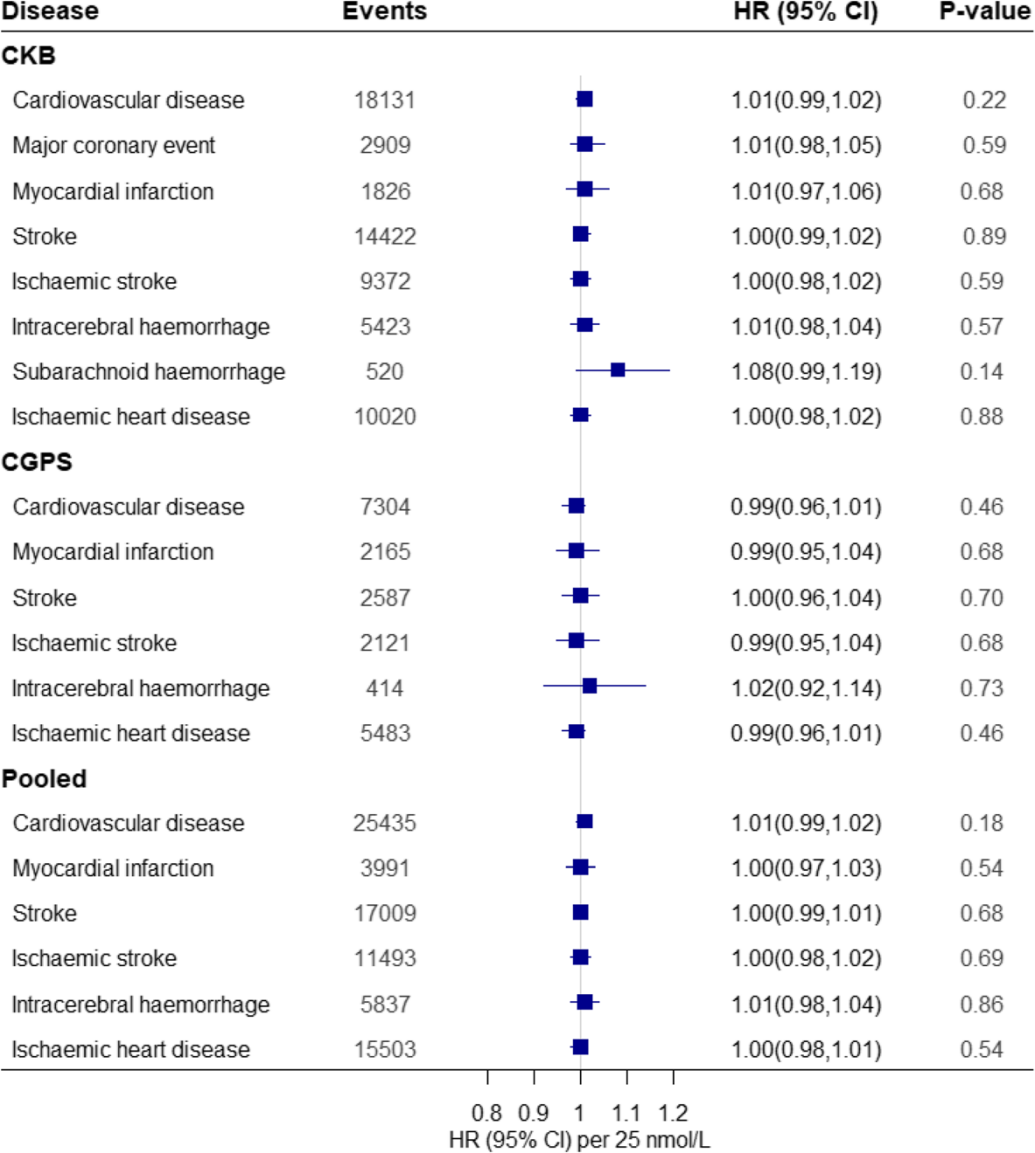
Instrumental variable estimates for vascular diseases. Analyses of two-SNP score with vascular diseases were estimated using cox proportional hazard regression models. We used a two-SNP score as instrument to estimate the influence of a 25 nmol/L increase in 25(OH)D concentrations on risk of vascular diseases. We calculated instrumental variable estimates of genetically determined hazard ratios by using the Wald-type estimator, which involves taking the ratio of the gene-outcome log hazard ratios to the gene-exposure coefficient and then exponentiating to express it as a hazard ratio. Two-SNP score was calculated based on *DHCR7* + *CYP2R1*: rs12785878 + rs10741657 in CKB and *DHCR7* + *CYP2R1*: rs7944926 + rs10741657 in CGPS. The *r*^2^ between rs12785878 and rs7944926 is 0.87. All values are adjusted for age, sex, and season and stratified by region

**Fig. 3:**
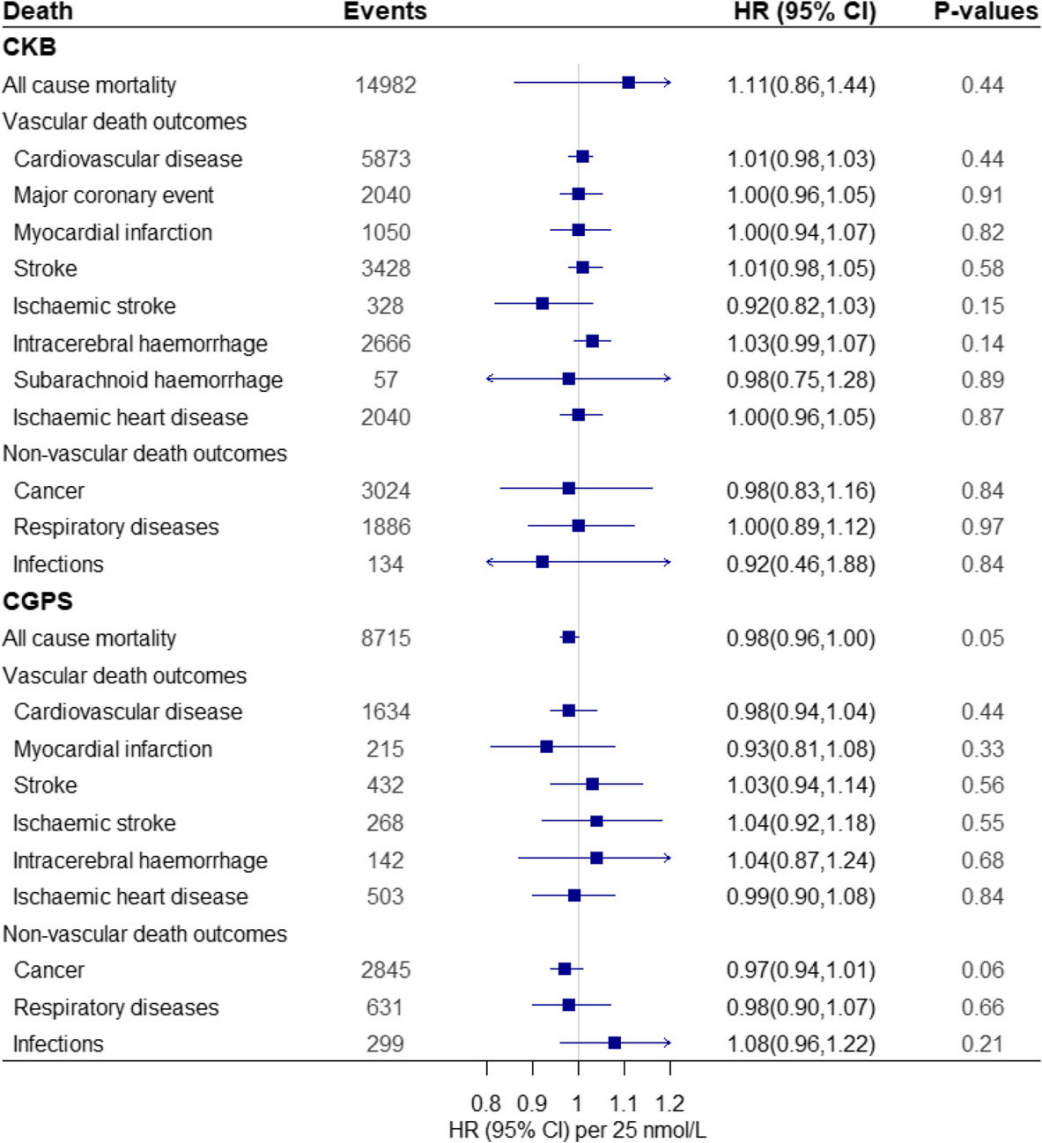
Instrumental variable estimates for mortality. Analyses of two-SNP score with mortality were estimated using cox proportional hazard regression models. We used a two-SNP score as instrument to estimate the influence of a 25 nmol/L increase in 25(OH)D concentrations on risk of mortality. We calculated instrumental variable estimates of genetically determined hazard ratios by using the Wald-type estimator, which involves taking the ratio of the gene-outcome log hazard ratios to the gene-exposure coefficient and then exponentiating to express it as a hazard ratio. Number of individuals in CKB and CGPS are 99,012 and 106,911, respectively. Two-SNP score was calculated based on *DHCR7* + *CYP2R1*: rs12785878 + rs10741657 in CKB and *DHCR7* + *CYP2R1*: rs7944926 + rs10741657 in CGPS. The *r*^2^ between rs12785878 and rs7944926 is 0.87. All values are adjusted for age, sex, and season and stratified by region

In both CKB and CGPS, there was no significant genetic association of the *DHCR7*/*CYP2R1* risk score with cause-specific vascular disease (Additional file 1: Tables S4 and S5) or mortality (Additional file 1: Table S6 and S7) after Bonferroni correction. Instrumental variable analysis did not show any significant causal association of genetically predicted 25(OH)D with any cause-specific vascular disease in CKB and CGPS (Fig. 2). Furthermore, genetically predicted 25(OH)D was not significantly associated with cause-specific mortality in CKB and CGPS (Fig. 3). However, the instrumental variable analysis indicated a marginal association for all-cause mortality per a 25 nmol/L higher in genetically predicted 25(OH)D concentrations (0.98 [0.96,1.00]) (Fig. 3). Sensitivity analyses yielded similar findings for genetically instrumented differences in 25(OH)D concentrations using the individual SNP or four-SNP score for each disease (Additional file 1: Table S4 to S7).

**Fig. 4:**
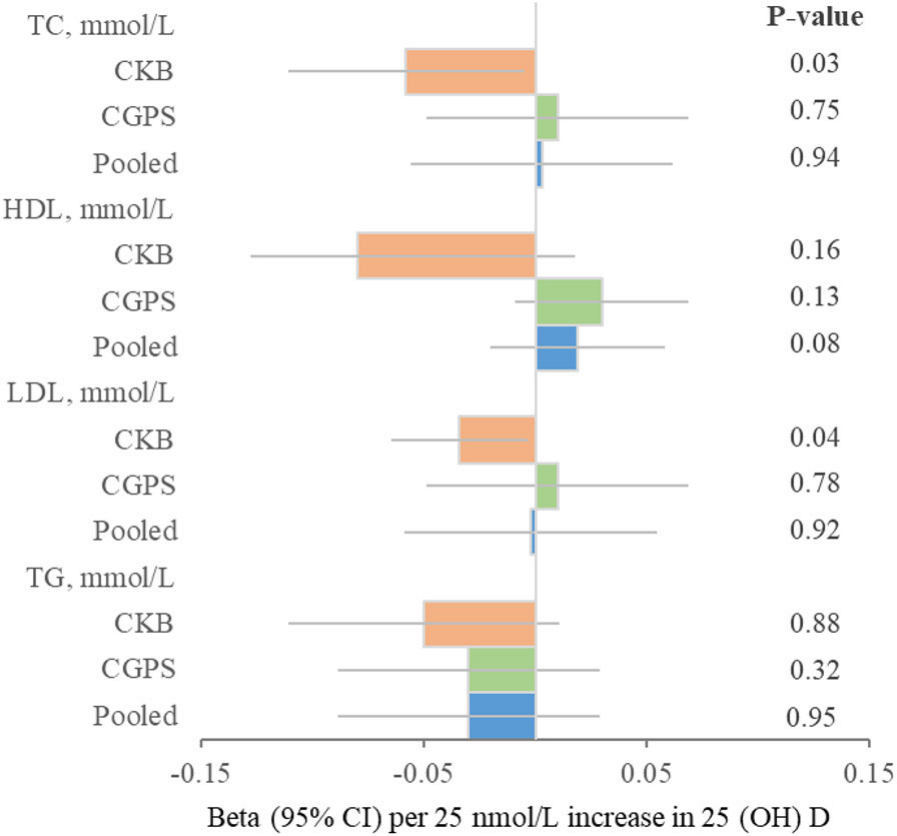
Instrumental variable estimates for lipids. Linear regression was also used to assess the associations of a two-SNP score with lipids. We used a two-SNP score as instrument to estimate the influence of a 25 nmol/L increase in 25(OH)D concentrations on lipids. We calculated instrumental variable estimates of genetically determined hazard ratios by using the Wald-type estimator, which involves taking the ratio of the gene-outcome coefficient to the gene-exposure coefficient. Two-SNP score was calculated based on *DHCR7* + *CYP2R1*: rs12785878 + rs10741657 in CKB and *DHCR7* + *CYP2R1*: rs7944926 + rs10741657 in CGPS. The *r*^2^ between rs12785878 and rs7944926 is 0.87. All values are adjusted for age, sex, and season and stratified by region

In addition, instrumental variable analyses showed significant associations with total and LDL cholesterol in CKB (Fig. 4 and Additional file 1: Table S8). Each 25 nmol/l genetic increase in 25(OH)D concentrations was associated with 0.058 mmol/L (0.22 mg/dL) and 0.034 mmol/L (0.13 mg/dL) lower total and LDL cholesterol in CKB. However, we did not observe causal associations for lipids and lipoproteins in CGPS and pooled cohorts (Fig. 4 and Additional file 1: Table S9).

## Discussion

The novel findings of this MR study involving 99,012 Chinese adults and 106,911 European adults suggest that genetically higher plasma 25(OH)D levels were not associated with lower risk of cause-specific vascular disease such as myocardial infarction, ischaemic heart disease, ischaemic stroke, stroke, intracerebral haemorrhage, and subarachnoid haemorrhage among both Chinese and European adults. Likewise, 25(OH)D may not influence lipid levels. Our results suggest that the inverse associations of vitamin D with cause-specific vascular disease and lipids could be the results of confounding.

Using an MR approach free from confounding and reverse causation, our study for the first time showed that genetically predicted 25(OH)D was not associated with intracerebral haemorrhage, subarachnoid haemorrhage, stroke, and ischaemic stroke, thus suggesting that vitamin D may not play a causal role in development of cause-specific vascular disease in Chinese or Europeans. The results suggest that individuals should be cautious about long-term supplementation with high doses of vitamin D3 [10]. The results are consistent with recent MR studies which did not show a causal effect of circulating vitamin D levels on risk of ischaemic heart disease or myocardial infarction [12], or cardiovascular mortality [13]. Findings from RCTs that do not show a protective effect of vitamin D3 supplementation on cardiovascular outcomes [9–11], supported our findings. However, previous RCTs did not specifically focus on vitamin D-deficient population. Thus, available evidence is not enough to support cardiovascular benefits or hazards of the commonly used vitamin D doses, but whether higher doses of vitamin D are beneficial is uncertain, or whether vitamin D supplementation has vascular effects in individuals with vitamin D deficiency remains unclear [9]. It worth noting that there are two major forms of vitamin D: vitamin D2 (ergocalciferol) and vitamin D3 (cholecalciferol). A meta-analysis demonstrated that vitamin D3 is more effective than vitamin D2 in maintaining circulating concentrations of 25(OH)D [28]. Previous randomised double-blind placebo-controlled trial examining the effects of vitamin D2 or D3 supplementation on cardiometabolic risk showed that both vitamin D2 or D3 did not influence blood pressure and cardiovascular risk, but decreased ApoB concentration and pulse wave velocity. Only vitamin D2 decreased total cholesterol [29]. However, so far, no RCT has investigated the benefits of supplemental vitamin D2 or D3 in preventing vascular diseases in Chinese adults. Whether vitamin D2 or D3 supplementation will be beneficial in patients with CVD or high-risk older patients is unknown and warrants further investigation.

Our findings could be explained by previous evidence that low 25(OH)D concentration is a marker of an unhealthy lifestyle or poor health [30], which has been associated with increased risk of vascular disease. Furthermore, previous MR studies suggest that cardiovascular factors [31], such as high remnant cholesterol, LDL cholesterol [32], and obesity [33], decrease plasma concentrations of 25(OH)D and increase vascular risk. That means low plasma 25(OH)D is a consequence of CVD rather than a causal factor. This could explain why we did not observe a causal association in MR analyses, despite strong observational associations in Europeans [4, 5]. In addition, a previous study showed that elevated parathyroid hormone levels identify a population with higher cardiovascular risk. Parathyroid hormone correlates to cardiovascular risk, but not vitamin D [34]. The potential mechanism is that parathyroid hormone induces oxidative stress and affects endothelial function [35]. Therefore, we have to acknowledge that the vitamin D-dependent parathyroid hormone, rather than vitamin D itself, might be a better predictor of cardiovascular risk. However, we have to acknowledge that the possibility of a non-linear association of vitamin D with cardiovascular outcomes cannot be excluded as it was not tested in the present MR study.

Although our MR analysis did not show causal effects of 25(OH)D on all-cause and cause-specific mortality in Chinese, results from Europeans demonstrated that 25(OH)D might be causally related to all-cause mortality and cancer mortality. Our findings are consistent with a European MR study, which showed that genetically reduced vitamin D concentrations were associated with increased all-cause, cancer mortality, but not with cardiovascular mortality [13]. Our results in Europeans are also supported by a meta-analysis of 56 RCTs mainly conducted in Europeans, which showed that vitamin D decreased all-cause and cancer mortality [11]. However, when different forms of vitamin D were assessed, only vitamin D3 (cholecalciferol) decreased mortality. Vitamin D2 (ergocalciferol) may even increase mortality [11]. It should be noted that recent European MR studies found evidence that genetically determined vitamin D concentrations were inversely associated with type 2 diabetes [36], and blood pressure and risk of hypertension [37]. These results could at least partly explain the reduced mortality among Europeans observed by us. However, so far, neither RCTs nor MR studies have investigated the causal effect of vitamin D on all-cause and cause-specific mortality in Chinese adults. Our present MR analysis for the first time documented that genetically elevated 25(OH)D was not associated with cause-specific vascular disease or mortality. These findings suggest possible ethnic differences in relationships between 25(OH)D levels and cancer mortality. A previous study showed that the effect of vitamin D3 supplementation on advanced colorectal adenomas varied according to vitamin D receptor genotypes [38]. Among the rs7968585AA genotype individuals (26%), supplemental vitamin D3 reduced risk by 64%, but increased risk by 41% among individuals with rs7968585 G alleles (74%) [38]. Therefore, whether benefits from vitamin D supplementation for the prevention of cancer vary according to vitamin D receptor genotype is unknown and warrants further investigation.

Previous RCTs have shown inconsistent results for the effects of vitamin D on plasma lipid levels [39–44]. However, it is worth noting that none of the RCTs were specifically conducted for evaluating the effect of supplemental vitamin D on lipids and lipoproteins. Interestingly, the largest trial (The WHI-Calcium+Vitamin D trial) conducted in 36,282 women showed that patients receiving combined vitamin D and omega-3 fatty acid supplementation had decreased serum very low-density lipoprotein cholesterol and serum triglycerides [42] while 400 IU vitamin D3 plus 1000 mg elemental calcium carbonate daily showed a decrease in LDL cholesterol after 6 weeks of intervention [43]. Consistently, our MR analysis in Chinese adults for the first time showed that each 25 nmol/l genetic increase in 25(OH)D concentration was associated with 0.058 mmol/L(0.22 mg/dL) and 0.034 mmol/L(0.13 mg/dL) lower total and LDL cholesterol, thus suggesting that 25(OH)D may play a causal role in lipid metabolism at least in Chinese adults. However, the potential mechanism warrants further investigation.

In the present MR analysis, we considered three assumptions to ensure the validity of our analyses [17, 18, 27]. First, our data demonstrated that the individual SNP and genetic score were associated with 25(OH)D (F statistic > 15), suggesting strong instrumental variables. Second, the instruments should be independent of measured and unmeasured confounders. Indeed, we found no association of genetic variants or score with measured confounders, thus satisfying an important condition for a valid MR experiment. Third, the genetic score should have no pleiotropic effect on the outcomes, which is an important consideration in MR studies. The third assumption may be violated by pleiotropy [45]. The SNPs encoding enzymes involved in the synthesis of 25(OH)D are believed to be more specific for 25(OH)D, but SNPs involved in the transport of 25(OH)D may have pleiotropic effects in addition to their effects on plasma 25(OH)D concentrations alone [31, 46, 47]. However, when we excluded SNPs involved in the transport of 25(OH)D, the association remained unchanged.

Several strengths of the present study merit consideration. This is the first MR analysis to examine the causal association of 25(OH)D with intracerebral haemorrhage and subarachnoid haemorrhage. This large MR study had a large number of disease events and high-quality 25(OH)D measurements by well-trained technicians and an appropriate design allowed us to gain sufficient power for estimation of causal effects. Another strength of our study was detailed information on known confounding factors, which allowed us to examine the three MR assumptions. Finally, we used standardised analytic methods to compare the differences in causal effects between Chinese and Europeans.

## Conclusion

In summary, this large-scale analysis provides reliable evidence that higher 25(OH)D may not play a causal role in developing cause-specific vascular disease in Chinese or Europeans. MR analysis further demonstrated that high vitamin D may not influence lipids.

## Additional file

Additional file 1: Supplemental tables providing additional information on genetic association with 25(OH)D, lipids and CVD. (DOCX 52 kb)

## Data Availability

All data generated or analysed during this study are included in this published article and its supplementary information files. Individual participant data are available at www.ckbiobank.org for researchers who meet the criteria for access to released data.

## Abbreviations

CCHS: Copenhagen City Heart Study
CGPS: Copenhagen General Population Study
CKB: China Kadoorie Biobank
GWAS: Genome-wide association studies
MR: Mendelian randomisation
RCT: Randomised intervention trial
SNP: Single nucleotide polymorphisms
