# Supplementary material for: Vitamin D and cause-specific vascular disease and mortality: a Mendelian randomisation study involving 99,012 Chinese and 106,911 European adults
Source: BMC Med. 2019 Aug 30;17:160. doi: 10.1186/s12916-019-1401-y (PMC6716818; doi:10.1186/s12916-019-1401-y)
Supplement: Supplementary file 1 — Supplemental tables providing additional information on genetic association with 25(OH)D, lipids and CVD. (DOCX 52 kb) [file 12916_2019_1401_MOESM1_ESM.docx]

**Supplemental materials**

Table S1. Genetic association with 25 (OH)D (nmol/L) in CKB and CGPS

|  | **Number of 25(OH)D increasing allele** | | | | |  |  |  |
| --- | --- | --- | --- | --- | --- | --- | --- | --- |
|  | **0** | **1** | **2** | **3** | **5~8** | **Per allele** | | **P** |
|  | **Mean(SE)** | **Mean(SE)** | **Mean(SE)** | **Mean(SE)** | **Mean(SE)** | **Beta(SE)** | **F value** |  |
| **CKB** |  |  |  |  |  |  |  |  |
| rs12785878 (*DHCR7*) | 62.67±0.53 | 63.63±0.39 | 65.44±0.62 | / | / | 1.488±0.382 | 282.78 | <0.0001 |
| rs10741657 (*CYP2R1*) | 63.35±0.80 | 63.67±0.42 | 64.09±0.44 | / | / | 0.341±0.396 | 277.85 | 0.389 |
| rs6013897(*CYP24A1*) | 63.91±0.34 | 63.60±0.56 | 64.45±1.74 | / | / | 0.039±0.516 | 272.89 | 0.94 |
| rs2282679 (*GC/DBP*) | 57.23±0.80 | 61.63±0.41 | 67.01±0.43 | / | / | 5.100±0.407 | 335.7 | <0.0001 |
| Two SNP score (DHCR7 + CYP2R1: rs12785878+rs10741657) | 62.46±1.70 | 62.53±0.61 | 63.56±0.46 | 64.48±0.51 | 65.51±0.97 | 0.954±0.276 | 278.53 | 0.001 |
| Four-SNP score | 58.97±1.51 | 59.62±0.85 | 62.25±0.55 | 64.80±0.40 | 67.79±0.93 | 1.839±0.208 | 294.25 | <0.0001 |
| **CGPS** |  |  |  |  |  |  |  |  |
| rs7944926 (*DHCR*7) | 52.13 (0.483) | 54.71 (0.224) | 56.49 (0.219) | / | / | 2.023±0.229 | 79.65 | 1.1e-18 |
| rs11234027 (*DHCR7*) | 52.20 (0.739) | 54.06 (0.263) | 56.11 (0.186) | / | / | 2.011±0.263 | 63.61 | 1.9e-14 |
| rs12794714 (*CYP2R1*) | 51.45 (0.363) | 54.89 (0.213) | 57.71 (0.253) | / | / | 3.074±0.214 | 206.82 | 1.5e-46 |
| rs10741657 (*CYP2R1*) | 52.90 (0.256) | 55.94 (0.212) | 58.09 (0.357) | / | / | 2.668±0.214 | 151.62 | 1.4e-35 |
| rs2282679 (*GC/DBP*)* | 42.49 (0.897) | 44.57 (0.393) | 48.68 (0.348) | / | / | 3.540±0.395 | 80.56 | 4.1e-19 |
| Two-SNP score (DHCR7 + CYP2R1: rs7944926+rs10741657) | 50.14 (0.833) | 52.49 (0.338) | 54.69 (0.237) | 57.29 (0.270) | 59.48 (0.526) | 2.383±0.157 | 234.07 | 4.1e-52 |
| Two-SNP score (DHCR7 + CYP2R1: rs7944926+rs12794714) | 49.50 (1.156) | 51.62 (0.432) | 53.57 (0.259) | 56.54 (0.241) | 59.16 (0.371) | 2.589±0.156 | 276.04 | 2.4e-61 |
| Four-SNP score | 50.68 (0.500) | 53.16 (0.267) | 55.58 (0.346) | 56.56 (0.299) | 59.32 (0.356) | 2.000±0.118 | 289.25 | 1.7e-64 |

Data are presented as Mean (SE). All values are adjusted for age, sex, season, and stratified by region. *Genotypes only avalable in 11732 individuals

Table S2. Association of genetic variants with confounders in CKB

|  | **rs12785878** | | | **P for trend** | **rs10741657** | | | **P for trend** | **rs6013897** | | | **P for trend** | **rs2282679** | | | **P for trend** |
| --- | --- | --- | --- | --- | --- | --- | --- | --- | --- | --- | --- | --- | --- | --- | --- | --- |
|  | **GG** | **GT** | **TT** |  | **GG** | **AG** | **AA** |  | **TT** | **AT** | **AA** |  | **GG** | **GT** | **TT** |  |
| Demographic factors |  |  |  |  |  |  |  |  |  |  |  |  |  |  |  |  |
| Age. mean (SD).y | 53.1±13.2 | 52.2±12.1 | 53.4±13.8 | 0.68 | 52.2±12.5 | 53.1±11.2 | 51.5±12.3 | 0.57 | 51.6±11.4 | 52.0±12.2 | 53.3±11.7 | 0.627 | 53.1±13.2 | 51.4±12.5 | 53.0±13.2 | 0.726 |
| Male. % | 48.21 | 48.79 | 47.87 | 0.73 | 48.9 | 48.57 | 46.26 | 0.62 | 48.49 | 48.45 | 47.47 | 0.682 | 49.32 | 48.1 | 48.49 | 0.781 |
| Urban region. % | 34.2 | 30.48 | 27.56 | 0.78 | 31.55 | 30.47 | 30.41 | 0.67 | 31.3 | 30.37 | 29.07 | 0.737 | 30.19 | 29.59 | 32.18 | 0.836 |
| Socioeconomic factors. % |  |  |  |  |  |  |  |  |  |  |  |  |  |  |  |  |
| Household income>20.000 yuan/year. % | 32.43 | 32.52 | 30.76 | 0.22 | 32.7 | 32.53 | 31.89 | 0.11 | 32.81 | 31.59 | 33.22 | 0.121 | 34.37 | 32.75 | 31.96 | 0.22 |
| High school education and above. % | 36.04 | 36.77 | 35.66 | 0.59 | 36.27 | 36.16 | 36.88 | 0.48 | 36.67 | 35.76 | 33.22 | 0.528 | 35.07 | 36.08 | 36.69 | 0.627 |
| Married. % | 86.64 | 86.86 | 86.25 | 0.49 | 86.47 | 86.79 | 86.86 | 0.38 | 86.8 | 86.29 | 84.85 | 0.418 | 85.65 | 86.66 | 86.83 | 0.517 |
| Lifestyle factors. % |  |  |  |  |  |  |  |  |  |  |  |  |  |  |  |  |
| Mean MET (SD) | 18.30±0.14 | 18.64±0.11 | 18.91±0.16 | 0.22 | 18.55±0.12 | 18.54±0.11 | 18.96±0.21 | 0.11 | 18.60±0.09 | 18.58±0.14 | 18.68±0.45 | 0.121 | 18.67±0.25 | 18.73±0.12 | 18.47±0.11 | 0.22 |
| Weekly alcohol consumption. (%) | 15.81 | 15.64 | 15.12 | 0.59 | 15.54 | 15.38 | 16.35 | 0.48 | 15.5 | 15.85 | 14.59 | 0.528 | 15.68 | 15.76 | 15.39 | 0.627 |
| Current smoking. (%) | 31.7 | 32.06 | 31.99 | 0.49 | 32.32 | 32.08 | 30.22 | 0.38 | 31.96 | 32.24 | 30.98 | 0.418 | 31.68 | 32.34 | 31.67 | 0.517 |
| Physical and blood measurements. mean (SD) |  |  |  |  |  |  |  |  |  |  |  |  |  |  |  |  |
| BMI. kg/m2 | 23.27±0.04 | 23.31±0.03 | 23.30±0.04 | 0.94 | 23.27±0.03 | 23.25±0.03 | 23.52±0.06 | 0.83 | 23.33±0.02 | 23.23±0.04 | 23.19±0.12 | 0.913 | 23.25±0.07 | 23.27±0.03 | 23.32±0.03 | 0.957 |
| SBP.mmHg | 137.70±0.25 | 138.59±0.19 | 138.97±0.29 | 0.48 | 138.12±0.21 | 138.67±0.20 | 138.40±0.38 | 0.37 | 138.44±0.16 | 138.49±0.26 | 137.57±0.82 | 0.407 | 138.95±0.44 | 138.35±0.21 | 138.37±0.20 | 0.506 |
| DBP.mmHg | 79.53±0.13 | 80.02±0.10 | 80.10±0.15 | 0.45 | 79.59±0.11 | 80.11±0.11 | 80.12±0.20 | 0.34 | 79.98±0.09 | 79.75±0.14 | 79.58±0.42 | 0.374 | 79.93±0.23 | 79.90±0.11 | 79.88±0.10 | 0.473 |

Continued

|  | **Two SNP score (DHCR7 + CYP2R1:rs12785878+rs10741657)** | | | | | | **Four SNP score** | | | | | |
| --- | --- | --- | --- | --- | --- | --- | --- | --- | --- | --- | --- | --- |
|  | **0** | **1** | **2** | **3** | **4** | **P** | **0~1** | **2** | **3** | **4~5** | **6~8** | **P** |
| Demographic factors |  |  |  |  |  |  |  |  |  |  |  |  |
| Age. mean (SD).y | 51.3±11.9 | 52.7±12.6 | 53.1±12.2 | 51.8±11.2 | 51.7±12.1 | 0.8151 | 51.6±12.4 | 53.1±12.2 | 51.6±13.1 | 52.2±12.0 | 53.0±13.2 | 0.8211 |
| Male. % | 46.2 | 48.52 | 47.93 | 49.13 | 48.78 | 0.8701 | 49.21 | 48.37 | 48.15 | 48.71 | 47.9 | 0.8761 |
| Urban region. % | 33.05 | 32.63 | 31.16 | 30.43 | 26.86 | 0.9251 | 30.97 | 32.38 | 31.42 | 30.95 | 28.51 | 0.9311 |
| Socioeconomic factors. % |  |  |  |  |  |  |  |  |  |  |  |  |
| Household income>20.000 yuan/year. % | 32.88 | 33.18 | 32.91 | 32.23 | 30.44 | 0.3091 | 35.36 | 32.88 | 33.42 | 32.48 | 29.63 | 0.3151 |
| High school education and above. % | 36.81 | 36.67 | 35.99 | 36.68 | 35.42 | 0.7161 | 36.26 | 36.61 | 36.31 | 36.62 | 34.61 | 0.7221 |
| Married. % | 85.65 | 87.6 | 86.39 | 86.66 | 86.19 | 0.6061 | 85.7 | 87.08 | 86.83 | 86.59 | 85.98 | 0.6121 |
| Lifestyle factors. % |  |  |  |  |  |  |  |  |  |  |  |  |
| Mean MET (SD) | 19.25±0.40 | 18.66±0.17 | 18.24±0.12 | 18.77±0.14 | 19.11±0.25 | 0.3091 | 18.99±0.47 | 18.80±0.24 | 18.48±0.15 | 18.54±0.10 | 18.86±0.23 | 0.3151 |
| Weekly alcohol consumption. (%) | 17.08 | 16.36 | 15.03 | 15.56 | 15.53 | 0.7161 | 16.1 | 16.43 | 15.97 | 15.34 | 14.99 | 0.7221 |
| Current smoking. (%) | 31.26 | 31.45 | 31.76 | 32.13 | 33.39 | 0.6061 | 32.09 | 31.54 | 31.9 | 32.18 | 31.97 | 0.6121 |
| Physical and blood measurements. mean (SD) |  |  |  |  |  |  |  |  |  |  |  |  |
| BMI. kg/m2 | 23.41±0.11 | 23.39±0.05 | 23.25±0.03 | 23.27±0.04 | 23.30±0.07 | 0.9801 | 23.26±0.12 | 23.22±0.06 | 23.37±0.04 | 23.29±0.03 | 23.21±0.06 | 0.9861 |
| SBP.mmHg | 138.44±0.73 | 138.12±0.30 | 138.33±0.22 | 138.47±0.25 | 139.22±0.46 | 0.5951 | 138.06±0.81 | 137.76±0.43 | 138.50±0.28 | 138.69±0.19 | 137.85±0.42 | 0.6011 |
| DBP.mmHg | 79.92±0.38 | 79.99±0.16 | 79.78±0.12 | 79.99±0.13 | 79.82±0.23 | 0.5621 | 80.11±0.41 | 79.57±0.22 | 79.99±0.15 | 80.07±0.10 | 79.20±0.21 | 0.5681 |

MET=metabolic equivalent of task.

Table S3. Association of genetic variants with confounders in CGPS

|  | **rs7944926 (*DHCR7*)** | | | | **rs10741657 (*CYP2R1*)** | | | |
| --- | --- | --- | --- | --- | --- | --- | --- | --- |
|  | **GG** | **GT** | **TT** | **P** | **GG** | **AG** | **AA** | **P** |
| Number of individuals | 50247 | 46129 | 10535 |  | 18723 | 51957 | 36231 |  |
| Demographic factors |  |  |  |  |  |  |  |  |
| Age (SD), years | 58.1 (13.1) | 58.0 (13.1) | 58.0 (13.1) | 0.57 | 58.1 (13.2) | 58.0 (13.1) | 58.0 (13.1) | 0.28 |
| Women, No. % | 27556 (55) | 25453 (55) | 5774 (55) | 0.62 | 10187 (54) | 28559 (55) | 20037 (55) | 0.05 |
| Lifestyle factors, % |  |  |  |  |  |  |  |  |
| Low leisure time physical activity | 3121 (6) | 2906 (6) | 627 (6) | 0.67 | 1205 (6) | 3186 (6) | 2263 (6) | 0.57 |
| Current smoker, No. % | 8522 (17) | 7955 (17) | 1846 (18) | 0.11 | 3249 (17) | 8802 (17) | 6272 (17) | 0.8 |
| Current drinker, No. % | 44829 (89) | 41014 (89) | 9403 (89) | 0.48 | 16710 (89) | 46246 (89) | 32290 (89) | 0.8 |
| Physical and blood measurements, mean (SD) |  |  |  |  |  |  |  |  |
| BMI, kg/m^2^ | 26.1 (4.3) | 26.1 (4.3) | 26.2 (4.3) | 0.38 | 26.2 (4.3) | 26.2 (4.3) | 26.1 (4.3) | 0.12 |
| SBP,mmHg | 141.6 (21.4) | 141.5 (21.4) | 141.8 (21.5) | 0.83 | 141.5 (21.0) | 141.6 (21.3) | 141.6 (21.6) | 0.62 |
| DBP,mmHg | 84.2 (11.5) | 84.2 (11.4) | 84.4 (11.4) | 0.37 | 84.1 (11.4) | 84.3 (11.4) | 84.3 (11.6) | 0.24 |
| Month of recruitment | 6.4 (3.4) | 6.4 (3.4) | 6.5 (3.4) | 0.34 | 6.4 (3.4) | 6.4 (3.4) | 6.4 (3.4) | 0.92 |

Continued

|  | **rs11234027** | | | | **rs12794714** | | | |
| --- | --- | --- | --- | --- | --- | --- | --- | --- |
|  | **TT** | **AT** | **AA** | **P** | **GG** | **GT** | **TT** | **P** |
| Number of individuals | 68326 | 34189 | 4396 |  | 36875 | 51953 | 18083 |  |
| Demographic factors |  |  |  |  |  |  |  |  |
| Age (SD), years | 58.1 (13.1) | 58.0 (13.1) | 57.9 (13.0) | 0.39 | 58.1 (13.1) | 58.0 (13.1) | 58.0 (13.1) | 0.31 |
| Women, No. % | 37614 (55) | 18790 (55) | 2379 (54) | 0.36 | 20130 (55) | 28644 (55) | 10009 (55) | 0.06 |
| Lifestyle factors, % |  |  |  |  |  |  |  |  |
| Low leisure time physical activity | 4199 (6) | 2216 (6) | 239 (5) | 0.67 | 2295 (6) | 3268 (6) | 1091 (6) | 0.53 |
| Current smoker, No. % | 11581 (17) | 5941 (17) | 801 (18) | 0.01 | 6318 (17) | 8871 (17) | 3134 (17) | 0.67 |
| Current drinker, No. % | 60934 (89) | 30405 (89) | 3907 (89) | 0.21 | 32949 (89) | 46226 (89) | 16071 (89) | 0.05 |
| Physical and blood measurements, mean (SD) |  |  |  |  |  |  |  |  |
| BMI, kg/m^2^ | 26.1 (4.3) | 26.2 (4.3) | 26.2 (4.3) | 0.19 | 26.2 (4.3) | 26.1 (4.3) | 26.1 (4.2) | 0.19 |
| SBP,mmHg | 141.5 (21.3) | 141.7 (21.5) | 142.0 (21.8) | 0.12 | 141.4 (21.0) | 141.6 (21.5) | 141.7 (21.7) | 0.6 |
| DBP,mmHg | 84.2 (11.5) | 84.3 (11.5) | 84.3 (11.4) | 0.36 | 84.2 (11.4) | 84.3 (11.4) | 84.3 (11.6) | 0.35 |
| Month of recruitment | 6.4 (3.4) | 6.4 (3.4) | 6.5 (3.4) | 0.68 | 6.4 (3.4) | 6.4 (3.4) | 6.4 (3.4) | 0.95 |

Continued

|  | **Two SNP score (DHCR7 + CYP2R1: rs7944926+rs10741657))** | | | | | | **Four SNP score** | | | | | |
| --- | --- | --- | --- | --- | --- | --- | --- | --- | --- | --- | --- | --- |
|  | **0** | **1** | **2** | **3** | **4** | **P** | **0~1** | **2** | **3** | **4~5** | **6~8** | **P** |
| Number of individuals | 8861 | 32216 | 41564 | 20806 | 3464 |  | 18721 | 26098 | 19212 | 33479 | 9401 |  |
| Demographic factors |  |  |  |  |  |  |  |  |  |  |  |  |
| Age (SD), years | 58.1 (13.2) | 58.1 (13.1) | 58.0 (13.1) | 58.0 (13.1) | 57.7 (13.0) | 0.24 | 58.1 (13.2) | 58.2 (13.1) | 57.9 (13.1) | 58.1 (13.1) | 57.7 (13.1) | 0.2 |
| Women, No. % | 4831 (55) | 17624 (55) | 22901 (55) | 11500 (55) | 1927 (56) | 0.08 | 10216 (55) | 14336 (55) | 10559 (55) | 18463 (55) | 5209 (55) | 0.14 |
| Lifestyle factors, % |  |  |  |  |  |  |  |  |  |  |  |  |
| Low leisure time physical activity | 561 (6) | 1981 (6) | 2639 (6) | 1279 (6) | 194 (6) | 0.47 | 1125 (6) | 1661 (6) | 1213 (6) | 2106 (6) | 549 (6) | 0.95 |
| Current smoker, No. % | 1489 (17) | 5483 (17) | 7159 (17) | 3576 (17) | 616 (18) | 0.2 | 3163 (17) | 4446 (17) | 3240 (17) | 5834 (17) | 1640 (17) | 0.08 |
| Current drinker, No. % | 7925 (89) | 28746 (89) | 36929 (89) | 18542 (89) | 3104 (90) | 0.51 | 16743 (89) | 23272 (89) | 17181 (89) | 29661 (89) | 8389 (89) | 0.03 |
| Physical and blood measurements, mean (SD) |  |  |  |  |  |  |  |  |  |  |  |  |
| BMI, kg/m^2^ | 26.2 (4.3) | 26.2 (4.3) | 26.1 (4.3) | 26.2 (4.3) | 26.1 (4.3) | 0.59 | 26.2 (4.3) | 26.1 (4.3) | 26.1 (4.3) | 26.2 (4.3) | 26.1 (4.2) | 0.58 |
| SBP,mmHg | 141.5 (21.1) | 141.5 (21.2) | 141.5 (21.4) | 141.6 (21.5) | 141.9 (22.0) | 0.83 | 141.4 (21.0) | 141.5 (21.3) | 141.5 (21.3) | 141.6 (21.5) | 141.8 (21.9) | 0.66 |
| DBP,mmHg | 84.1 (11.5) | 84.3 (11.4) | 84.2 (11.5) | 84.4 (11.5) | 84.4 (11.5) | 0.14 | 84.2 (11.4) | 84.2 (11.5) | 84.3 (11.4) | 84.3 (11.5) | 84.4 (11.6) | 0.26 |
| Month of recruitment | 6.4 (3.4) | 6.4 (3.4) | 6.4 (3.4) | 6.4 (3.4) | 6.6 (3.4) | 0.56 | 6.4 (3.4) | 6.4 (3.4) | 6.4 (3.4) | 6.4 (3.4) | 6.5 (3.4) | 0.93 |

MET=metabolic equivalent of task.

Data are presented as mean(SD) for continous variables, and n% for catigorical variables.

Table S4. Adjusted Hazard Ratios (aHRs) for vascular disease per 25(OH)D increasing allele in CKB

| **Diseases** | **No. participants** | | **rs12785878 (*DHCR7*)** | **rs10741657 (*CYP2R1*)** | **rs6013897(*CYP24A1*)** | **rs2282679 (*GC/DBP*)** | **Four-snp score** |
| --- | --- | --- | --- | --- | --- | --- | --- |
|  | **Cases** | **Individuals** | **HR (95% CI)** | **HR (95% CI)** | **HR (95% CI)** | **HR (95% CI)** | **HR (95% CI)** |
|  |  |  | **per allele** | **per allele** | **per allele** | **per allele** | **per allele** |
| Major vascular event | 18,131 | 80,881 | 1.01(0.99,1.03) | 1.00(0.98,1.02) | 1.00(0.97,1.03) | 0.98(0.96,1.00) | 1.00(0.99,1.01) |
| Major coronary event | 2,909 | 96,103 | 1.02(0.97,1.08) | 1.00(0.95,1.06) | 1.05(0.98,1.13) | 0.94(0.89,0.99) | 1.00(0.97,1.03) |
| Myocardial infarction | 1,826 | 97,186 | 1.04(0.97,1.11) | 0.98(0.92,1.05) | 1.04(0.95,1.14) | 0.94(0.88,1.01) | 1.00(0.96,1.04) |
| Stroke | 14,422 | 84,590 | 1.01(0.99,1.04) | 0.99(0.97,1.02) | 1.00(0.97,1.04) | 0.98(0.96,1.01) | 1.00(0.99,1.01) |
| Ischaemic stroke | 9,372 | 89,640 | 1.01(0.98,1.04) | 0.99(0.96,1.02) | 1.01(0.97,1.05) | 0.98(0.95,1.01) | 1.00(0.98,1.01) |
| Intracerebral haemorrhage | 5,423 | 93,589 | 1.02(0.98,1.06) | 1.00(0.96,1.04) | 1.00(0.95,1.05) | 1.00(0.96,1.04) | 1.00(0.98,1.03) |
| Subarachnoid | 520 | 98,492 | 1.11(0.99,1.26) | 1.05(0.93,1.20) | 0.98(0.83,1.15) | 1.06(0.93,1.21) | 1.06(0.99,1.13) |
| Ischaemic heart disease | 10,020 | 88,992 | 1.00(0.97,1.03) | 1.00(0.97,1.02) | 0.99(0.95,1.03) | 0.98(0.95,1.01) | 0.99(0.98,1.01) |

All values are adjusted for age, sex, season, and stratified by region.

Table S5. Adjusted Hazard Ratios (aHRs) for vascular disease per 25(OH)D increasing allele in CGPS

| **Diseases** | **No. individuals** | | **rs7944926 (DHCR7)** | **rs10741657 (*CYP2R1*)** | **rs11234027** | **rs12794714** | **rs2282679 (*GC*)*** | **Two-snp score (DHCR7 + CYP2R1: rs7944926+rs12794714))** | **Four-snp score** |
| --- | --- | --- | --- | --- | --- | --- | --- | --- | --- |
|  | **Cases** | **Participants** | **HR (95% CI)** | **HR (95% CI)** | **HR (95% CI)** | **HR (95% CI)** | **HR (95% CI)** | **HR (95% CI)** | **HR (95% CI)** |
|  |  |  | **per allele** | **per allele** | **per allele** | **per allele** | **per allele** | **per allele** | **per allele** |
| CVD | 7,304 | 99,595 | 0.97(0.94,1.01) | 1.00(0.97,1.03) | 0.98(0.94,1.02) | 1.01(0.98,1.04) | 1.01(0.93,1.10) | 0.99(0.97,1.02) | 0.99(0.98,1.01) |
| Myocardial infarction | 2,165 | 104,674 | 1.00(0.94,1.07) | 0.99(0.93,1.05) | 0.98(0.91,1.06) | 1.01(0.95,1.07) | 0.96(0.84,1.10) | 1.00(0.96,1.05) | 1.00(0.96,1.03) |
| Stroke | 2,587 | 105,488 | 0.98(0.93,1.04) | 1.02(0.97,1.08) | 0.96(0.90,1.03) | 1.04(0.98,1.10) | 1.14(0.99,1.31) | 1.01(0.97,1.05) | 1.00(0.97,1.03) |
| Ischaemic stroke | 2,121 | 105,737 | 0.97(0.91,1.03) | 1.02(0.96,1.08) | 0.95(0.88,1.02) | 1.04(0.98,1.11) | 1.12(0.96,1.32) | 1.01(0.96,1.05) | 1.00(0.96,1.03) |
| Intracerebral haemorrhage | 414 | 106,729 | 1.06(0.91,1.23) | 0.99(0.86,1.14) | 1.06(0.90,1.26) | 0.99(0.86,1.15) | 1.36(0.96,1.91) | 1.02(0.92,1.14) | 1.01(0.94,1.10) |
| Ischaemic heart disease | 5,483 | 100,756 | 0.97(0.93,1.01) | 1.00(0.96,1.04) | 0.98(0.94,1.03) | 1.01(0.97,1.05) | 0.95(0.87,1.04) | 0.99(0.96,1.02) | 1.00(0.97,1.02) |

All values are adjusted for age, sex, season, and region.

*Number of all individuals for GC rs2282679: 11726. Number of cases for GC rs2282679: CVD, 1399; Myocardial infarction, 481, Stroke, 503, Ischaemic stroke, 405, Intracerebral haemorrhage, 82, Ischaemic heart disease, 1059.

CVD includes all the following diagnoses: Myocardial infarction, Stroke, ischaemic stroke, intracerebral haemorrhage, ischaemic heart disease

Table S6. Adjusted Hazard Ratios (aHRs) for mortality per 25(OH)D increasing allele in CKB

| **Death** | **No. participants** | | **rs12785878 (*DHCR7*)** | **rs10741657 (*CYP2R1*)** | **rs6013897(*CYP24A1*)** | **rs2282679 (*GC/DBP*)** | **Four-snp score** |
| --- | --- | --- | --- | --- | --- | --- | --- |
|  | **Cases** | **Participants** | **HR (95% CI)** | **HR (95% CI)** | **HR (95% CI)** | **HR (95% CI)** | **HR (95% CI)** |
|  |  |  | **per allele** | **per allele** | **per allele** | **per allele** | **per allele** |
| Major vascular event | 5,873 | 93,139 | 1.02(0.98,1.06) | 1.00(0.96,1.03) | 1.02(0.98,1.08) | 0.98(0.95,1.02) | 1.00(0.99,1.02) |
| Major coronary event | 2,040 | 96,972 | 1.00(0.94,1.07) | 1.00(0.94,1.07) | 1.03(0.95,1.12) | 0.92(0.86,0.98) | 0.99(0.95,1.02) |
| Myocardial infarction | 1,050 | 97,962 | 1.02(0.94,1.11) | 0.99(0.90,1.08) | 1.02(0.90,1.14) | 0.94(0.85,1.03) | 0.99(0.94,1.04) |
| Stroke | 3,428 | 95,584 | 1.04(0.99,1.09) | 0.98(0.94,1.03) | 1.01(0.95,1.08) | 1.01(0.96,1.07) | 1.01(0.99,1.04) |
| Ischaemic stroke | 328 | s | 0.95(0.81,1.11) | 0.89(0.76,1.05) | 1.11(0.90,1.38) | 1.00(0.84,1.18) | 0.97(0.89,1.05) |
| Intracerebral haemorrhage | 2,666 | 96,346 | 1.06(1.00,1.12) | 1.00(0.95,1.06) | 1.01(0.94,1.09) | 1.02(0.97,1.09) | 1.03(1.00,1.06) |
| Subarachnoid | 57 | 98,955 | 0.80(0.55,1.15) | 1.24(0.84,1.85) | 1.39(0.79,2.44) | 0.99(0.67,1.48) | 1.04(0.85,1.27) |
| Ischaemic heart disease | 2,040 | 96,972 | 1.00(0.94,1.07) | 1.00(0.94,1.07) | 1.03(0.95,1.12) | 0.92(0.86,0.98) | 0.99(0.95,1.02) |

All values are adjusted for age, sex, season, and stratified by region.

Table S7. Adjusted Hazard Ratios (aHRs) for genetic association with mortality in CGPS

| **Mortality** | **No. participants** | | **rs7944926 *(DHCR7)*** | **rs10741657 (*CYP2R1*)** | **rs11234027** | **rs12794714** | **rs2282679 (*GC*)*** | **Two-snp score *(DHCR7* + *CYP2R1:* rs7944926+rs12794714))** | **Four-snp score** |
| --- | --- | --- | --- | --- | --- | --- | --- | --- | --- |
|  | **Cases** | **Participants** | **HR (95% CI)** | **HR (95% CI)** | **HR (95% CI)** | **HR (95% CI)** | **HR (95% CI)** | **HR (95% CI)** | **HR (95% CI)** |
|  |  |  | **per allele** | **per allele** | **per allele** | **per allele** | **per allele** | **per allele** | **per allele** |
| All cause mortality | 8,715 | 106,911 | 1.00(0.96,1.03) | 0.97(0.94,1.00) | 0.99(0.95,1.02) | 0.96(0.93,0.99) | 1.04(0.97,1.11) | 0.97(0.95,1.00) | 0.98(0.96,0.99) |
| **Vascular death outcomes:** |  |  |  |  |  |  |  |  |  |
| CVD | 1,634 | 106,911 | 1.01(0.94,1.09) | 0.96(0.90,1.03) | 1.02(0.93,1.11) | 1.00(0.93,1.07) | 1.05(0.90,1.23) | 1.00(0.95,1.05) | 0.99(0.95,1.03) |
| Myocardial infarction | 215 | 106,911 | 0.92(0.75,1.14) | 0.94(0.78,1.14) | 1.02(0.81,1.30) | 1.04(0.86,1.25) |  | 0.98(0.85,1.13) | 0.98(0.89,1.09) |
| Stroke | 432 | 106,911 | 1.05(0.90,1.21) | 1.02(0.89,1.17) | 1.08(0.91,1.28) | 1.06(0.92,1.22) |  | 1.06(0.96,1.17) | 1.03(0.95,1.11) |
| Ischaemic stroke | 268 | 106,911 | 1.01(0.84,1.22) | 1.07(0.90,1.27) | 1.05(0.84,1.32) | 1.11(0.93,1.33) |  | 1.06(0.93,1.21) | 1.04(0.94,1.15) |
| Intracerebral haemorrhage | 142 | 106,911 | 1.14(0.88,1.49) | 0.95(0.74,1.22) | 1.15(0.86,1.54) | 0.96(0.75,1.23) |  | 1.04(0.88,1.24) | 1.01(0.88,1.14) |
| Ischaemic heart disease | 503 | 106,911 | 1.02(0.89,1.18) | 0.96(0.84,1.08) | 1.06(0.91,1.24) | 0.98(0.87,1.11) | 1.00(0.76,1.31) | 1.00(0.91,1.10) | 0.99(0.93,1.07) |
| **Non-vascular death outcomes:** |  |  |  |  |  |  |  |  |  |
| Cancer | 2845 | 106,911 | 0.98(0.92,1.03) | 0.97(0.92,1.02) | 0.95(0.89,1.02) | 0.96(0.91,1.01) | 1.08(0.95,1.22) | 0.97(0.93,1.01) | 0.97(0.95,1.00) |
| Respiratory diseases | 631 | 106,911 | 1.04(0.92,1.17) | 0.93(0.83,1.05) | 0.98(0.86,1.13) | 0.91(0.82,1.02) | 1.14(0.89,1.47) | 0.97(0.89,1.05) | 0.97(0.90,1.03) |
| Infections | 299 | 106,911 | 1.12(0.93,1.34) | 1.05(0.89,1.24) | 1.10(0.90,1.34) | 1.10(0.93,1.29) | 1.15(0.76,1.72) | 1.11(0.98,1.26) | 1.06(0.97,1.16) |
| All other causes | 1,736 | 106,911 | 1.02(0.95,1.09) | 0.99(0.92,1.06) | 1.01(0.93,1.10) | 0.96(0.90,1.03) | 0.95(0.82,1.11) | 0.99(0.94,1.04) | 1.00(0.96,1.04) |

All values are adjusted for age, sex, season, and region.

*Number of all participants for GC rs2282679: 11732. Number of cases for GC rs2282679: All cause mortality, 1949, CVD, 437; Cancer , 632, Respiratory diseases, 156, Infections , 56, All other causes, 425.

Table S8. Genetic association with lipids level in CKB

| **SNP** | **rs12785878 (*DHCR7*)** | | | | | **rs10741657 (*CYP2R1*)** | | | | |
| --- | --- | --- | --- | --- | --- | --- | --- | --- | --- | --- |
|  | **GG** | **GT** | **TT** | **Per T allele** | **P** | **GG** | **AG** | **AA** | **Per A allele** | **P** |
|  | **Mean(SE)** | **Mean(SE)** | **Mean(SE)** | **Beta(SE)** |  | **Mean(SE)** | **Mean(SE)** | **Mean(SE)** | **Beta(SE)** |  |
| **Apoa, mg/dl** | 129.67±0.41 | 129.75±0.30 | 128.59±0.45 | -0.659±0.243 | 0.007 | 129.81±0.59 | 129.34±0.32 | 129.48±0.33 | -0.045±0.254 | 0.859 |
| **Apob, mg/dl** | 84.58±0.42 | 83.80±0.32 | 83.78±0.47 | -0.655±0.224 | 0.003 | 83.70±0.61 | 84.39±0.33 | 83.68±0.35 | -0.363±0.234 | 0.121 |
| **Lpa, mmol/L** | 36.63±0.91 | 36.97±0.71 | 35.92±1.06 | 0.124±0.495 | 0.802 | 37.60±1.43 | 37.63±0.73 | 35.22±0.77 | -1.373±0.517 | 0.008 |
| **TC, mmol/L** | 4.66±0.02 | 4.65±0.02 | 4.62±0.02 | -0.033±0.010 | 0.002 | 4.63±0.03 | 4.66±0.02 | 4.63±0.02 | -0.011±0.011 | 0.323 |
| **HDL, mmol/L** | 1.23±0.01 | 1.23±0.00 | 1.22±0.01 | -0.005±0.003 | 0.117 | 1.23±0.01 | 1.22±0.00 | 1.23±0.00 | -0.001±0.003 | 0.877 |
| **LDL, mmol/L** | 2.34±0.01 | 2.32±0.01 | 2.32±0.02 | -0.020±0.007 | 0.007 | 2.31±0.02 | 2.34±0.01 | 2.32±0.01 | -0.004±0.008 | 0.579 |
| **TG, mmol/L** | 1.99±0.03 | 2.04±0.03 | 1.99±0.03 | -0.008±0.017 | 0.637 | 2.01±0.04 | 2.03±0.03 | 2.00±0.03 | 0.005±0.018 | 0.78 |

Continued

| **SNP** | **rs6013897(*CYP24A1*)** | | | | | **rs2282679 (*GC/DBP*)** | | | | |
| --- | --- | --- | --- | --- | --- | --- | --- | --- | --- | --- |
|  | **TT** | **AT** | **AA** | **Per A allele** | **P** | **GG** | **GT** | **TT** | **Per T allele** | **P** |
|  | **Mean(SE)** | **Mean(SE)** | **Mean(SE)** | **Beta(SE)** |  | **Mean(SE)** | **Mean(SE)** | **Mean(SE)** | **Beta(SE)** |  |
| **Apoa, mg/dl** | 129.22±0.26 | 130.13±0.42 | 127.65±1.19 | 0.095±0.329 | 0.772 | 129.54±0.66 | 129.94±0.34 | 129.02±0.31 | -0.056±0.265 | 0.831 |
| **Apob, mg/dl** | 84.22±0.27 | 83.74±0.43 | 80.95±1.30 | -0.880±0.304 | 0.004 | 84.51±0.72 | 83.82±0.34 | 84.06±0.32 | 0.096±0.244 | 0.695 |
| **Lpa, mmol/L** | 36.60±0.58 | 36.56±1.00 | 38.57±3.15 | -0.481±0.672 | 0.474 | 34.28±1.48 | 36.63±0.75 | 37.07±0.74 | 0.591±0.539 | 0.273 |
| **TC, mmol/L** | 4.65±0.01 | 4.66±0.02 | 4.47±0.06 | -0.031±0.014 | 0.027 | 4.67±0.03 | 4.64±0.02 | 4.65±0.02 | 0.003±0.011 | 0.794 |
| **HDL, mmol/L** | 1.22±0.00 | 1.24±0.01 | 1.20±0.02 | 0.001±0.004 | 0.864 | 1.22±0.01 | 1.23±0.00 | 1.22±0.00 | 0.000±0.003 | 0.932 |
| **LDL, mmol/L** | 2.33±0.01 | 2.32±0.01 | 2.22±0.04 | -0.034±0.010 | 0.001 | 2.34±0.02 | 2.32±0.01 | 2.33±0.01 | 0.006±0.008 | 0.498 |
| **TG, mmol/L** | 2.01±0.02 | 2.03±0.03 | 1.97±0.09 | 0.003±0.023 | 0.892 | 2.03±0.06 | 2.00±0.03 | 2.02±0.02 | -0.001±0.018 | 0.978 |

Continued

| **SNP** | **Two-SNP score *(DHCR7* + *CYP2R1:* rs12785878+rs10741657))** | | | | | **Four-SNP score** | | | | | | |
| --- | --- | --- | --- | --- | --- | --- | --- | --- | --- | --- | --- | --- |
|  | **0** | **1** | **2** | **3** | **4** | **0~1** | **2** | **3** | **4~5** | **6~8** | **Per T allele** | **P** |
|  | **Mean(SE)** | **Mean(SE)** | **Mean(SE)** | **Mean(SE)** | **Mean(SE)** | **Mean(SE)** | **Mean(SE)** | **Mean(SE)** | **Mean(SE)** | **Mean(SE)** | **Beta(SE)** |  |
| **Apoa, mg/dl** | 128.93±1.08 | 129.83±0.49 | 129.91±0.35 | 128.95±0.38 | 128.91±0.70 | 129.62±1.12 | 130.12±0.69 | 129.92±0.44 | 129.19±0.30 | 128.94±0.64 | -0.214±0.134 | 0.111 |
| **Apob, mg/dl** | 84.72±1.22 | 84.04±0.50 | 84.47±0.37 | 83.52±0.40 | 83.48±0.76 | 84.77±1.33 | 84.82±0.71 | 84.54±0.47 | 83.64±0.31 | 83.53±0.70 | -0.408±0.124 | 0.001 |
| **Lpa, mmol/L** | 37.67±2.70 | 37.46±1.11 | 37.34±0.82 | 35.81±0.89 | 34.58±1.65 | 34.47±2.56 | 37.84±1.51 | 37.11±1.00 | 36.25±0.70 | 37.04±1.62 | -0.239±0.274 | 0.383 |
| **TC, mmol/L** | 4.63±0.05 | 4.65±0.02 | 4.68±0.02 | 4.62±0.02 | 4.62±0.04 | 4.63±0.06 | 4.69±0.03 | 4.66±0.02 | 4.63±0.01 | 4.62±0.03 | -0.017±0.006 | 0.003 |
| **HDL, mmol/L** | 1.21±0.02 | 1.23±0.01 | 1.23±0.01 | 1.22±0.01 | 1.23±0.01 | 1.23±0.02 | 1.23±0.01 | 1.23±0.01 | 1.23±0.00 | 1.22±0.01 | -0.001±0.002 | 0.44 |
| **LDL, mmol/L** | 2.33±0.04 | 2.33±0.02 | 2.34±0.01 | 2.31±0.01 | 2.32±0.03 | 2.33±0.04 | 2.37±0.02 | 2.34±0.02 | 2.32±0.01 | 2.32±0.02 | -0.011±0.004 | 0.007 |
| **TG, mmol/L** | 1.98±0.09 | 2.00±0.04 | 2.03±0.03 | 2.03±0.03 | 1.93±0.05 | 1.92±0.08 | 2.05±0.07 | 2.00±0.03 | 2.03±0.03 | 1.98±0.05 | 0.001±0.009 | 0.996 |

Data are presented as Mean (SE)

All values are adjusted for age, sex, season, and stratified by region.

Table S9. Genetic association with lipids level in CGPS

| **SNP** | **No. of individuals** | **rs7944926 (*DHCR7*)** | | | | | **rs11234027** | | | | |
| --- | --- | --- | --- | --- | --- | --- | --- | --- | --- | --- | --- |
|  |  | **TT** | **GT** | **GG** | **Per T allele** | **P** | **AA** | **AT** | **TT** | **Per T allele** | **P** |
|  |  | **Mean(SE)** | **Mean(SE)** | **Mean(SE)** | **Beta(SE)** |  | **Mean(SE)** | **Mean(SE)** | **Mean(SE)** | **Beta(SE)** |  |
| **TC, mmol/L** | 106911 | 5.58 (0.010) | 5.58 (0.005) | 5.58 (0.005) | 0.002 (0.005) | 0.7 | 5.56 (0.016) | 5.59 (0.006) | 5.58 (0.004) | 0.002 (0.006) | 0.77 |
| **HDL, mmol/L** | 106911 | 1.62 (0.005) | 1.62 (0.002) | 1.62 (0.002) | 0.001 (0.002) | 0.6 | 1.62 (0.007) | 1.62 (0.003) | 1.62 (0.002) | 0.004 (0.003) | 0.16 |
| **LDL, mmol/L** | 106911 | 3.22 (0.009) | 3.23 (0.004) | 3.23 (0.004) | 0.001 (0.004) | 0.75 | 3.20 (0.014) | 3.24 (0.005) | 3.23 (0.004) | 0.000 (0.005) | 0.97 |
| **TG, mmol/L** | 106911 | 1.68 (0.011) | 1.67 (0.005) | 1.67 (0.005) | -0.001 (0.005) | 0.88 | 1.70 (0.016) | 1.67 (0.006) | 1.67 (0.004) | -0.005 (0.006) | 0.35 |

Continued

| **SNP** | **No. of individuals** | **rs10741657 (*CYP2R1*)** | | | | | **rs12794714** | | | | |
| --- | --- | --- | --- | --- | --- | --- | --- | --- | --- | --- | --- |
|  |  | **GG** | **AG** | **AA** | **Per G allele** | **P** | **TT** | **GT** | **GG** | **Per T allele** | **P** |
|  |  | **Mean(SE)** | **Mean(SE)** | **Mean(SE)** | **Beta(SE)** |  | **Mean(SE)** | **Mean(SE)** | **Mean(SE)** | **Beta(SE)** |  |
| **TC, mmol/L** | 106911 | 5.58 (0.006) | 5.58 (0.005) | 5.58 (0.008) | 0.001 (0.005) | 0.88 | 5.58 (0.008) | 5.58 (0.005) | 5.58 (0.006) | -0.001 (0.005) | 0.86 |
| **HDL, mmol/L** | 106911 | 1.62 (0.002) | 1.62 (0.002) | 1.63 (0.003) | 0.004 (0.002) | 0.042 | 1.62 (0.004) | 1.62 (0.002) | 1.63 (0.002) | 0.005 (0.002) | 0.021 |
| **LDL, mmol/L** | 106911 | 3.23 (0.005) | 3.23 (0.004) | 3.23 (0.007) | -0.002 (0.004) | 0.72 | 3.23 (0.007) | 3.23 (0.004) | 3.23 (0.005) | -0.004 (0.004) | 0.29 |
| **TG, mmol/L** | 106911 | 1.68 (0.006) | 1.67 (0.005) | 1.67 (0.008) | -0.004 (0.005) | 0.36 | 1.68 (0.008) | 1.67 (0.005) | 1.68 (0.006) | -0.001 (0.005) | 0.77 |

Continued

| **SNP** | **rs2282679 (*GC*)** | | | | | **Two SNP score *(DHCR7* + *CYP2R1:* rs7944926+rs10741657))** | | | | |
| --- | --- | --- | --- | --- | --- | --- | --- | --- | --- | --- |
|  | **TT** | **GT** | **GG** | **Per T allele** | **P** | **0** | **1** | **2** | **3** | **4** |
|  | **Mean(SE)** | **Mean(SE)** | **Mean(SE)** | **Beta(SE)** |  | **Mean(SE)** | **Mean(SE)** | **Mean(SE)** | **Mean(SE)** | **Mean(SE)** |
| **TC, mmol/L** | 5.68 (0.035) | 5.72 (0.015) | 5.70 (0.014) | 0.000 (0.015) | 0.98 | 5.60 (0.018) | 5.58 (0.007) | 5.58 (0.005) | 5.58 (0.006) | 5.59 (0.011) |
| **HDL, mmol/L** | 1.63 (0.016) | 1.63 (0.007) | 1.62 (0.006) | -0.006 (0.007) | 0.38 | 1.63 (0.008) | 1.61 (0.003) | 1.62 (0.002) | 1.63 (0.003) | 1.63 (0.005) |
| **LDL, mmol/L** | 3.27 (0.031) | 3.29 (0.014) | 3.30 (0.012) | 0.011 (0.014) | 0.42 | 3.23 (0.016) | 3.23 (0.007) | 3.23 (0.005) | 3.23 (0.005) | 3.23 (0.010) |
| **TG, mmol/L** | 1.82 (0.041) | 1.82 (0.018) | 1.80 (0.016) | -0.013 (0.018) | 0.48 | 1.69 (0.018) | 1.67 (0.008) | 1.68 (0.005) | 1.66 (0.006) | 1.68 (0.012) |

Continued

| **SNP** | **Two SNP score *(DHCR7* + *CYP2R1:* rs7944926+rs12794714))** | | | | | | **Four SNP score** | | | | | | |
| --- | --- | --- | --- | --- | --- | --- | --- | --- | --- | --- | --- | --- | --- |
|  | **0** | **1** | **2** | **3** | **4** | **P** | **0~1** | **2** | **3** | **4~5** | **6~8** | **Per T allele** | **P** |
|  | **Mean(SE)** | **Mean(SE)** | **Mean(SE)** | **Mean(SE)** | **Mean(SE)** |  | **Mean(SE)** | **Mean(SE)** | **Mean(SE)** | **Mean(SE)** | **Mean(SE)** | **Beta(SE)** |  |
| **TC, mmol/L** | 5.58 (0.025) | 5.59 (0.009) | 5.58 (0.006) | 5.58 (0.005) | 5.59 (0.008) | 0.89 | 5.60 (0.011) | 5.57 (0.006) | 5.58 (0.008) | 5.57 (0.007) | 5.59 (0.008) | 0.001 (0.003) | 0.74 |
| **HDL, mmol/L** | 1.63 (0.011) | 1.62 (0.004) | 1.62 (0.003) | 1.62 (0.002) | 1.63 (0.004) | 0.041 | 1.62 (0.005) | 1.62 (0.003) | 1.62 (0.003) | 1.62 (0.003) | 1.63 (0.003) | 0.003 (0.001) | 0.0059 |
| **LDL, mmol/L** | 3.22 (0.023) | 3.24 (0.008) | 3.23 (0.005) | 3.23 (0.005) | 3.23 (0.007) | 0.58 | 3.24 (0.010) | 3.23 (0.005) | 3.23 (0.007) | 3.22 (0.006) | 3.23 (0.007) | -0.001 (0.002) | 0.64 |
| **TG, mmol/L** | 1.67 (0.026) | 1.68 (0.010) | 1.67 (0.006) | 1.67 (0.005) | 1.68 (0.008) | 0.75 | 1.69 (0.011) | 1.67 (0.006) | 1.68 (0.008) | 1.66 (0.007) | 1.67 (0.008) | -0.002 (0.003) | 0.36 |

Data are presented as Mean (SE)

All values are adjusted for age, sex, season, and stratified by region.

*Genotypes only avalable in 11732 individuals
